# The Chemistry and Physics of Bayfol^®^ HX Film Holographic Photopolymer

**DOI:** 10.3390/polym9100472

**Published:** 2017-09-26

**Authors:** Friedrich-Karl Bruder, Thomas Fäcke, Thomas Rölle

**Affiliations:** Covestro Deutschland AG, Building D201, D-51365 Leverkusen, Germany; thomas.faecke@covestro.com (T.F.); thomas.roelle@covestro.com (T.R.)

**Keywords:** photopolymer, volume holographic optical element, photochemistry, interference recording, mass manufacturing

## Abstract

Holographic photopolymers are a new technology to create passive diffractive optical elements by a pure laser interference recording. In this review, we explain the chemistry concepts of light harvesting in an interference pattern and the subsequent grating formation as chemical response. Using the example of the newly developed Bayfol^®^ HX film we discuss the reaction-diffusion driven photo-polymerization process for an index modulation formation to create volume phase gratings. Further we elucidate the selection of monomer chemistry and discuss details of the recording conditions based on the concept of exposure dosage and exposure time. Influences ranging from high dosage recording to low power recording are explained and how to affect the desired diffraction efficiency. Finally, we outline and demonstrate the process to mass manufacturing of volume phase gratings.

## 1. Introduction

In human history, the ability to control light had a fundamental impact on how we interact and work with each other and with our environment. From the control of the fire to Edison’s light bulb to modern light emitting (laser) diodes the creation of light and managing it is fundamental to our life. For a long time, passive optical elements were based only on refraction and reflection. Diffractive optical elements remained in niche applications like scientific instruments. This is due to the fact that manufacturing is labor intense and their fragility prevented a wider use in mainstream applications.

We describe here the technology of holographic photopolymers that enable a much wider and more flexible use to manage light by engineering specific volume holographic optical elements (vHOEs). Those materials, specifically when they are made available in film format, push forward the boundaries that diffractive optics exhibited in the past. Photopolymer in general are materials that are sensitized to react upon light with a chemical response. Holographic photopolymers record an interference pattern thus exhibits chemical formation of diffractive gratings. A specific benefit to the ones described herein are those that do not require any chemical and thermal post processing, which is of specific benefit for the creation of optical components. Mass manufacturing becomes easier and precise and drives forward the adoption in the consumer space. Their chemical basis as plastic products links them to classic plastic processing to reach out into popular post hologram recording conversion techniques.

The basic chemical concept, the mechanics of the diffractive grating formation as chemical response in those new products made available as Bayfol^®^ HX films (Covestro AG, Leverkusen, Germany) is discussed. Key aspects of influences on the grating formation as function of the spatial frequency (*SF*), the exposure dosage and the exposure power are explained. We discuss the photochemistry that is used and how this influences the photon yield. After the initial chemical response we describe the influence of the photo-polymerization on the formation of index modulation of the phase grating including proper selection of polymerizing monomers (usually called writing monomers).

Holographic recording is done with an interference setup, where a coherent laser light source is split and illuminates from two angles the photopolymer film which is the recording medium. This concept is universal and can be used for individual holographic optical element recordings, while also specific master-to-copy setups are discussed. As the last step in this pure photonic recording process we explain the bleaching process of these photopolymers and give an insight into digital holographic recording.

### Volume Holographic Optical Elements and Their Applications

vHOEs are thin and lightweight film optics, where the film type influences the optical function. In contrast to surface gratings vHOEs exhibit an angular and spectral selectivity [1] that can be chosen by the thickness of the photopolymer film layer. By this selectivity the optics designer can chose the “On Bragg” condition where the recorded optical function is operative. In contrast no change to the wave front is observed in all other angles/wavelengths (so called “Off Bragg” conditions). Combined with the high transparency in the Off Bragg condition vHOEs are an enabler to new key components like optical combiners for augmented reality glasses [2], large head-up displays [3], and transparent screens [4]. They can be used as embedded stacked optics for spectrometers [5], sensors and additional functionalities in existing display technologies as well as new backlight devices for future display technologies [6,7]. They can be engineered to be ideal for applications where customized filter functions are needed, e.g., for laser protection [8]. Due to the inherent nature to create complex wave fronts, vHOEs can also be used as well in the design of luminaires, e.g., for automotive lighting [9]. In large area applications like new projection display solutions vHOEs can help to improve daylight projection contrast [10] and can be used to collect light more efficiently for solar cells [11,12]. A classical home turf for vHOEs is the security market, where their visual effects are a key for recognition without being obtrusive to the product. From banknotes to ID card to brand protection uses, a wide field of industrial and consumer applications are in development.

Holographic photopolymers are essential for many future optic designs and complement other technology developments like light emitting (laser) diodes, spatial light modulators and mobile computing in compact designs. vHOEs are always linked to full optical system designs, so their spectral and angular selectivity has to be considered. Advanced vHOEs comprise spatial and/or spectral multiplexing, which means that several vHOEs can be integrated in one single recording media film. This ability will drive their adoption into the consumer electronics space. Unique is the ability to design the diffraction efficiency in wide ranges from sub percent level to close to 100%. In this review, we discuss the details of this holographic photopolymer technology which is another example of a beautiful marriage between physics and chemistry.

## 2. Photopolymers as Recording Media for vHOEs

The need for an easy to use, size scalable recording medium prescribes material properties that in turn determine the necessary chemistry. A suitable recording medium must not require pre- or post-processing, has to provide high transparency and low haze after recording and will allow a high diffractive performance. Additionally, the unrecorded medium has to be manufactured at high quality standards and the recorded vHOE can be applied in mass production assembly lines. This property profile can be realized e.g., with a web coated film product.

Several approaches to provide such a material have been reported in the last decades from industrial as well as from academic laboratories. A major motivation for holographic recording material developments has been the field of optical data storage and several material concepts have been described and reviewed elsewhere [13]. While the fundamental physical principles for the holographic recording of a vHOE or a data page have subtle but significant deviations, the material design has to focus more densely on the material class that can realize the requirements. For good reasons, well designed photopolymers are able to fulfill these requirements.

### 2.1. Working Principle of a 2-Chemistry Photopolymer Film

For recording a stable optical hologram function, the recording medium has to have a certain minimum mechanical modulus to enable a proper grating formation. Liquid recording media tend to experience shrinkage issues that lead to unacceptable optical aberrations. In simple photopolymer systems, a part of the writing chemistry has to be consumed by flood exposure before the interference pattern recording to generate this minimum modulus. By this, a significant part of the dynamic range is already consumed and moreover due to the pre-exposure the whole recording process becomes less reliable.

Matrix formation can be realized by two reactive chemistries that do not interfere with each other (orthogonal chemistry), so that the writing chemistry has to be unaffected by this matrix formation [14,15].

A very important advantage of the 2-chemistry approach is the high miscibility of all liquid precursors ahead of matrix formation. After matrix formation, no de-mixing of the writing chemistry occurs when carefully designing the components. After hologram recording and curing of the writing chemistry a stable interpenetrating network between matrix and writing chemistry is formed [16].

**Figure 1:**
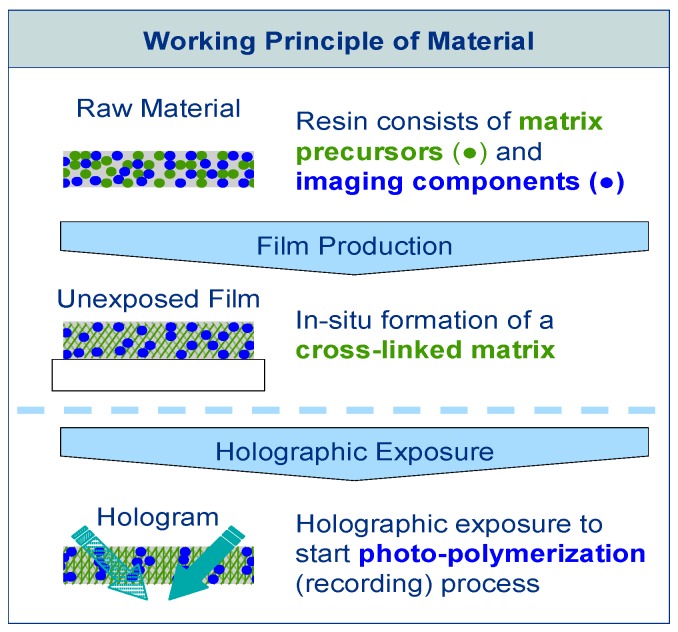
Working principle of Bayfol^®^ HX film photopolymer [17]: Matrix precursors and imaging components form the raw material resin. The matrix precursors are cross-linked to form a stable matrix system hosting the imaging components during film production. This film product is stable until exposed to light. The film is exposed to suitable light to initiate the photo-polymerization that forms the hologram structure during the recording process.

The raw material of Bayfol^®^ HX film type photopolymer is composed of two different chemistries, as is shown in Figure 1. The matrix precursors, which are cross-linked thermally during the manufacturing process forming a polymer network and determining the physical stability of the photopolymer, compose the first chemistry. The imaging components compose the second chemistry. These imaging components are responsible for the hologram formation during exposure. They include functionalities for the photo-initiation of the polymerization and polymerizable groups (monomers). With fully formed matrix, the unexposed photopolymer can be stored, shipped and handled easily as long as safe-light conditions are used to prevent accidental photo-polymerization. By exposing the photopolymer with a laser irradiation interference pattern, causing spatially distributed photo-polymerization, the hologram is recorded. During the exposure photo-polymerization is initiated in the bright fringes of the laser light interference pattern by activating neighboring imaging components. During the exposure the polymerizable monomers diffuse within the matrix until they react with an activated monomer or polymer chain. A refractive index modulation in the material that represents the hologram is caused by the process of polymerization in the bright regions and net diffusion away from the dark regions. For complete hologram formation no further processing is needed. Photo-curing is only needed to fully bleach the absorbing species to gain optimal transparency and ensuring that the writing chemistry has been fully consumed.

By this reaction-diffusion mechanism a pure phase hologram is generated throughout the thickness *d* of the photo reactive layer. In the simplest case of recording the interference pattern of two plane waves a sinusoidal modulation of the refractive index represents this phase grating. The amplitude of this sinusoidal refractive index modulation is in the following referred to ∆*n*_1_ and represents the performance of the photopolymer under certain recording geometries and recording conditions [17].

One example of a typical Bayfol^®^ HX film grade for research and development purposes that was studied in [18] is an acrylate based 2-chemistry photopolymer material and its typical composition is described in [19]. Such a class of photopolymer can be used for many applications comprising the fabrication of optical lenses, mirrors, filters, waveguides, diffraction elements and more complex 3D holographic images. Owing to the high values of refractive index modulation and thus achievable diffraction efficiency it shows great potential as versatile holographic media. The described composition in [19] was designed to achieve high diffraction efficiency at ~50 μm film thickness for reflection recording geometries. This specific photopolymer film has its strengths in applications where a high spatial frequency and/or high angle and spectral selectivity are required.

### 2.2. Web-Coating of Bayfol^®^ HX Film

The 2-chemistry approach allows a direct web-coating on a transparent thermoplastic carrier film (substrate). In a dryer station the thermoset matrix is cured without affecting the writing chemistry. It is obvious that the coating has to be done in a safe light environment, meaning low brightness and a selected spectrum to assure that the writing chemistry does not start to polymerize. Photopolymer layer thicknesses of a few µm up to >50 µm can be coated on transparent substrates of thicknesses ranging from about 20 µm up to >100 µm. Transparent film substrates are based on polyethylene terephthalate (PET), polycarbonate (PC), cellulosic triacetate (TAC) and amorphous polyamide (PA). After curing the photopolymer matrix, the surface of the photopolymer layer is protected with a removable cover layer. Usually this cover layer is polyethylene (PE) or one of the above-mentioned substrates as well. After de-lamination of the protective film, the inherent tack of the unrecorded photopolymer allows excellent optical and mechanical coupling to the recording support surface. As mentioned, the cover layer can also be chosen from high quality optical films that remain on the photopolymer surface during hologram recording. In this case, proper optical and mechanical coupling has to be assured by other means.

## 3. Modelling the Recording Mechanism of Bayfol^®^ HX Film

Zhao and Mouroulis [20] as well as Noiret et al. [21] for the first time described in 1994 the holographic recording mechanism in dry photopolymers by the model of photo-polymerization driven diffusion (PDD). In this PDD model the local monomer consumption rate was set proportional to the local intensity of the interference field. This means ideal polymerization kinetics is assumed in the PDD model and moreover the recording mechanism is set to be local in time and space. Despite these simplifying assumptions the PDD model is able to predict the fact that the achievable index modulation ∆*n*_1_ can drop off significantly if the spatial frequency (*SF*) of a recorded holographic grating in a dry photopolymer becomes small. This phenomenon is usually addressed as the low spatial frequency cutoff.

However many recording materials also experience a high spatial frequency cutoff, which limits its possible resolution. PDD is not able to predict this high spatial frequency cutoff. For example due to the finite size of the silver grains in silver halide recording materials there is a limit to the resolution and by this a high spatial-frequency cutoff. In dry photopolymers no such particles or precipitates exist. Therefore the reason for an observed high spatial frequency cutoff must be different. By describing the reaction as a non-local process in time and space the concept of non-local photo-polymerization driven diffusion (NPDD) was introduced by Sheridan et al. [22]. One channel for non-locality could be, due to the formation of extended polymer chains or networks, the propagation of the reactive macro-radical in space away from its original position where the chain growth was initiated. A further channel for non-locality could stem from the primary radicals formed in the photo-initiation system. If those primary radicals (or very short polymer chains) are too small and of too low reactivity they may diffuse too fast and start photo-polymerization spatially displaced from the position where the primary radical was generated. By ascribing a finite resolution length σ, characteristic for the specific photopolymer system, the NPDD model is able to predict the high spatial frequency cutoff. Within the relevant visible range of *SF* (<7500 1/mm corresponding to a Bragg mirror grating for λ = 400 nm in a medium with refractive index *n* = 1.5) high resolution photopolymers should have no high spatial-frequency cutoff to be able to record bright and full-color reflection holograms.

Also implementation of various model improvements with respect to the relation between monomer consumption rate and local intensity was conducted. For example the relation was changed from a linear one to a square root behavior [23] by adapting steady state conditions. An extension to a general power law [24] with an exponent γ in the range of 0.5 to 1 was done in a later stage. Absorption effects have been included also [25].

Gleeson and Sheridan have given a general review of the work done for free-radical photo-polymerization in holographic recording in [26]. For limiting recording situations like saturation recording, short pulse recording and multiplexing general analytical expressions are reported by Sheridan et al. [27].

In [17] we outlined the saturation recording behavior for the PDD case referring to the scheme given by Kelly et al. [24]. It is straightforward to extend this to the NPDD case. By assuming that only the non-reacted monomers are able to move over larger distances or longer times, as done in the above mentioned reaction-diffusion models, the diffusion equation for the volume fraction of the non-reacted monomer φ*_Mo_* can be formulated as:

(1) $$\frac{\partial \varphi_{Mo}}{\partial t}=D\cdot \frac{\partial^{2}\varphi_{Mo}}{\partial x^{2}}-\kappa \cdot P^{\gamma }\cdot \varphi_{Mo}\cdot {\left(1+V\cdot \cos\left(K\cdot x\right)\right)}^{\gamma }$$

For the non-reacted monomer a constant diffusion coefficient *D* is assumed. The incident recording power is denoted as *P*. The various details of the photo-polymerization are summarized in the constant κ [24]. The wave number of the recorded one dimensional grating is *K* = 2π/Λ = 2π⋅*SF* and related to the inverse grating spacing Λ. The modulation depth of the interference pattern, also called fringe visibility is denoted as *V*.

As we have shown in [17], for saturation recording Δ*n*_1_ is only a function of the system parameter *S*, characterizing the ratio of the reaction time scale τ*_R_* over the diffusion time scale τ*_D_*.

(2) $$\Delta n_{1}\left(S\right)=\Delta n_{1}\left(\frac{K^{2}\cdot D}{\kappa \cdot P^{\gamma }}\right)=\Delta n_{1}\left(\frac{4\pi^{2}\cdot SF^{2}\cdot D}{\kappa \cdot P^{\gamma }}\right)$$

Equation (2) suggests a scaling behavior. That means a power response curves of Δ*n*_1_ taken at different *SF* can be combined (shifted) to a master power response curve by appropriate recalibration of *P* for each *SF*. The parameter γ can be identified from the respective (shift) factor α via:

(3) $$\alpha =\frac{P_{1}}{P_{2}}={\left(\frac{K_{1}}{K_{2}}\right)}^{2/\gamma }$$

**Figure 2:**
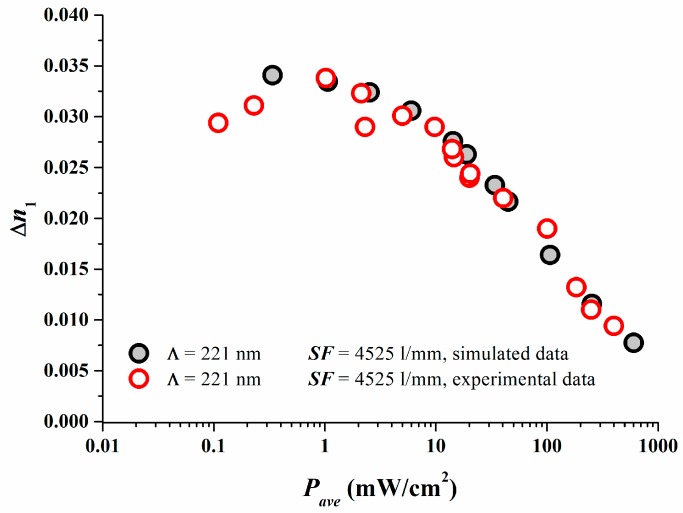
Comparison of the experimental (open symbols) and simulated (filled symbols) mastered power response curve after proper calibration [17].

The excellent match between reaction-diffusion model predictions and experimental results are depicted in Figure 2 in which the properly calibrated simulations results are compared to the experimental data [17].

As shown in [17] we set κ*_ex_* = 1.0 cm^2γ^/(s·mW^γ^) for simplicity and identified *D_ex_* = 2.5 × 10^−10^ cm^2^/s in accordance to what was found in [18]. A comparison of the simulated dosage response curve ∆*n*_1_ versus *E_ave_* can be done after having now γ and equivalent (*D_ex_*/κ*_ex_*) estimated for the photopolymer and the properly calibrated model. The following scaling for the dosage between simulation and experiment can be deduced (note *S* is kept the same between simulation and experiment):

(4) $$E_{ave}=E\cdot \frac{P_{ave}}{P}\cdot \frac{D}{D_{ex}}$$

**Figure 3:**
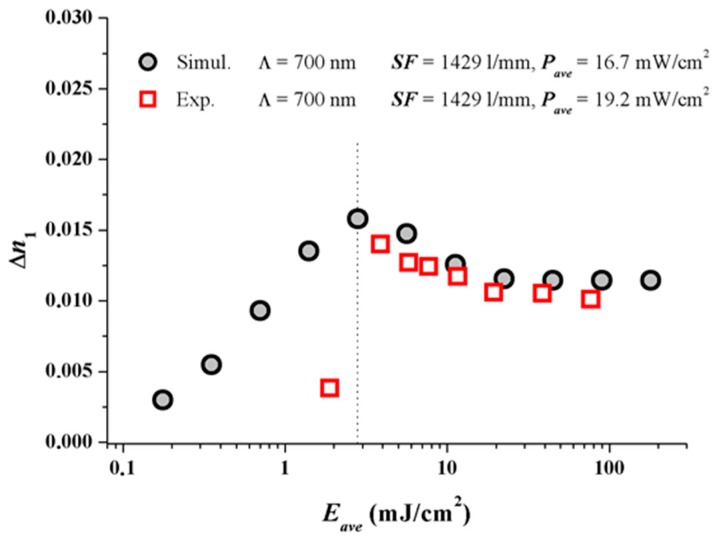
Comparison of dosage response curves for simulated data and experimental data for similar recording power densities *P_ave_* [17]. Note for the same *P_ave_* simulation and experiment should coincide.

Figure 3 shows the results. At larger dosages after the peak ∆*n*_1_ value in the simulation has been achieved, obviously the experimental dosage response coincides very well with the predicted dosage response. As in real systems always oxygen is present. Oxygen quenches radicals and inhibits the onset of photo-polymerization and therefore the onset of grating formation. One very obvious reason for the discrepancy at small dosages may stem from the fact that oxygen is not included in our simple model so far.

As mentioned above the inclusion of non-local reaction diffusion effects can be included in our simple model. Following Kelly et al. [24] Equation (1) has to be extended to:

(5) $$\frac{\partial \varphi_{Mo}}{\partial t}=D\cdot \frac{\partial^{2}\varphi_{Mo}}{\partial x^{2}}-\int_{-\infty }^{\infty }\kappa \cdot P^{\gamma }\cdot \varphi_{Mo}\cdot {\left(1+V\cdot \cos\left(K\cdot x\prime \right)\right)}^{\gamma }\cdot \frac{1}{\sqrt{2\pi \cdot \sigma^{2}}}\cdot \exp\left(-\frac{{\left(x-x\prime \right)}^{2}}{2\cdot \sigma^{2}}\right)\cdot dx\prime$$

For *V* equal to 1 and in the case of saturation recording we then find ∆*n*_1_ = ∆*n*_1_(*S*; *K*^2^⋅σ^2^). The high spatial frequency cutoff is described here as *K*^2^⋅σ^2^. Now ∆*n*_1_ starts to drop down due to insufficient resolution of the photopolymer if Λ = 1/*SF* is reduced and approaches the same order of magnitude as σ. We conducted respective model calculations for *P_ave_* = 8 mW/cm^2^ with varying non-locality σ^2^ using the identified system parameters γ, (*D_ex_*/κ*_ex_*) and the identified calibration of ∆*n*_1_.

**Figure 4:**
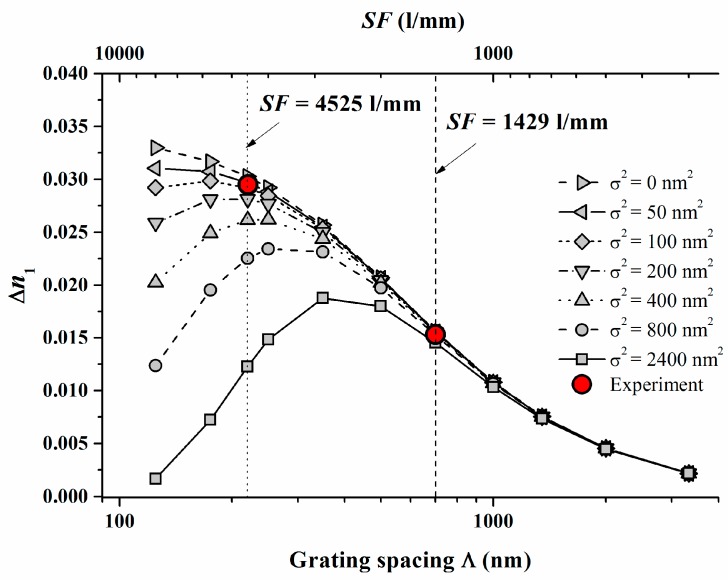
Calculated ∆*n*_1_ versus Λ = 1/*SF* for varying non-local parameters σ^2^ and *P_ave_* = 8 mW/cm^2^. The filled symbols indicate the respective experimental values measured at *SF* = 1429 L/mm (dashed line) and *SF* = 4525 L/mm (dotted line) [17].

The following Figure 4 shows the results with the two experimental ∆*n*_1_ values added which were deduced from plane wave plane wave recordings [17] at *P_ave_* = 8 mW/cm^2^.

In Figure 4 the dashed and the dotted lines refer to the respective *SF* = 1429 1/mm and 4525 1/mm. We can conclude from Figure 4 that σ^2^ should be in the range of <100 nm^2^. For a variety of photopolymers σ^2^ values are estimated in [22] and the smallest values cited are larger than 2000 nm^2^. These results underline that for Bayfol^®^ HX we do not have to expect a significant drop of ∆*n*_1_ due to a high spatial frequency cutoff at all *SF* relevant for visible light (e.g., for RGB reflection holograms *SF* ~ 4000–7500 1/mm).

**Figure 5:**
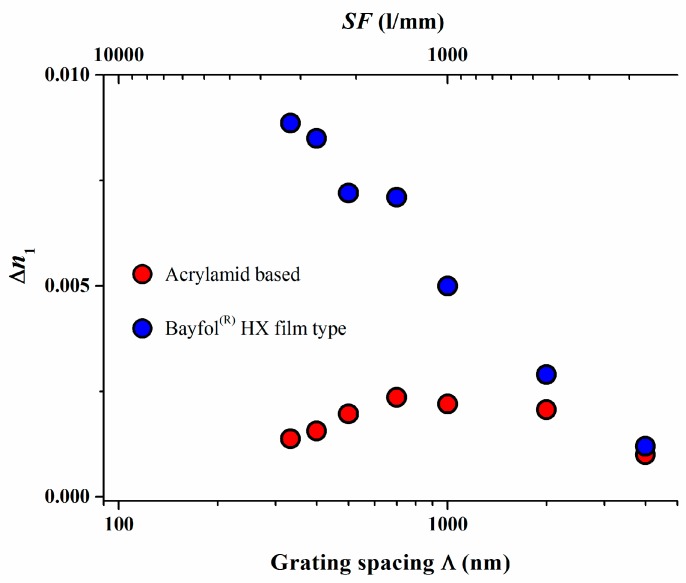
Experimental values of ∆*n*_1_ (symbols) versus grating spacing Λ (or spatial frequency *SF* ~ 1/Λ) for experimental Bayfol^®^ HX film type photopolymers in comparison to an acrylamid based photopolymer system [18]. Extracted values for σ^2^ are 85 nm^2^ for the experimental Bayfol^®^ HX film type photopolymer and 3840 nm^2^ for the acrylamid based photopolymer.

The absence of a high spatial frequency cutoff was further confirmed in a study in which a Bayfol^®^ HX film type photopolymer was compared to the widely studied acrylamide based photopolymer with respect to their experimentally measured and simulated spatial frequency response [18]. The results are depicted in Figure 5.

Again for the experimental Bayfol^®^ HX film type photopolymer a value for σ^2^ of 85 nm^2^ could be extracted whereas a σ^2^ of 3840 nm^2^ was found for the acrylamid based photopolymer. To relate these numbers to a grating spacing Λ of a retro-reflecting Bragg mirror for λ = 400 nm in a medium with a refractive index *n* of 1.5 which is 133 nm, the non-locality σ in a Bayfol^®^ HX film type photopolymer is in the range of 9–10 nm which is still much smaller than 133 nm. In an acrylamid based photopolymer this smearing σ of the localization of the interference pattern is in the range of 62 nm which almost half of the respective grating spacing. This large σ explains why in such a type of photopolymer the recording of a high spatial frequency grating shows a very low index modulation ∆*n*_1_.

### Recording at the Low Spatial Frequency Cutoff

At a first glance the drop of ∆*n*_1_ with decreasing *SF* or increasing *P*, due to the low spatial frequency cut off may appear as drawback for photopolymers. However this ∆*n*_1_ drop was used very successfully to record high quality vHOE gratings in Bayfol^®^ HX film type photopolymers. Those transmission gratings are used for spectral analysis in astronomical telescopes [28]. For transmission gratings over-modulation can cause difficulties in managing the optical performance of a vHOE in practical recording. This is due to the fact that the product ∆*n*_1_⋅*d* and by this the diffraction efficiency that is proportional to sin^2^ (Constant⋅∆*n*_1_⋅*d*) may vary strongly with the recording dosage *E*. Constant represents the recording geometry. Therefore the dosage margin to hit the maximum diffraction efficiency can become very tight. Due to the variation of ∆*n*_1_ via *P* the specific material can be tuned easily to maximum diffraction efficiency for transmission gratings at ideal fringe visibility *V* for saturated dosage *E*. This results in largest dosage margins for the recording process and *V* close to 1 reveals lowest noise in the recorded grating.

## 4. Optimization of the Photo-Initiator System for the Visible Spectrum

For a photopolymer material used in holographic recording its spectral sensitivity has to be aligned to the wavelength λ of laser light used for recording to enable a high efficiency of the conversion of the photon’s energy resulting in an overall economically feasible recording step. Due to the limited power of highly coherent lasers, holographic recording costs are heavily influenced by the overall efficiency of the photonic steps. This overall efficiency is determined by the molecular absorption coefficient of the sensitizer dyes at the recording wavelength λ, the quantum yield with which the reactive species are formed and react, the reactivity of generated photo products, and the possibility of the bleaching of the sensitizer dyes during the photo-polymerization [13,29,30,31,32]. The photo-polymerization efficiency thus depends on the nature of photo-initiators. Photo-initiating systems for the visible spectrum that were developed in recent years include ketocoumarines, functional dyes, thioxanthones, bis-acylphosphine oxides, pyrylium and thiopyrylium salts, (cationic) dyes in combination with a borate salt, dyes/bis-imidazole derivatives/thiols, photosensitizer/chlorotriazine/additives, etc. [33,34]. This diverse offering of photo-active chemicals for phase hologram recording poses the task to select the best in class. Furthermore, a crucial necessity for the photo active substances is to do their job traceless, i.e., for any optical application the visible range, no remaining coloration, stain or even hue will be accepted by the end-user.

From a mechanistic approach, the photo-initiation of a radical polymerization can occur by a direct or a sensitized decomposition of the photo-initiator. According to this, photosensitive systems can be classified as one, two or even three-component systems, with the one-component system having all required functionalities being encoded in one single molecule and the three component system using three individual molecules to fulfill the task. From this simplistic point of view, the material development has either to prepare the perfect molecule or to select a smart combination of two [35] or three components [36]. Since there is good evidence for all these concepts, one example for each one- and two-component system is discussed first.

### 4.1. One-Component System

**Scheme 1:**
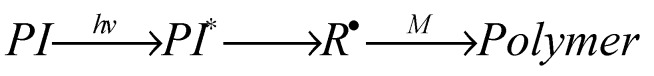
Reaction of a one-component photo-initiator system.

**Scheme 2:**
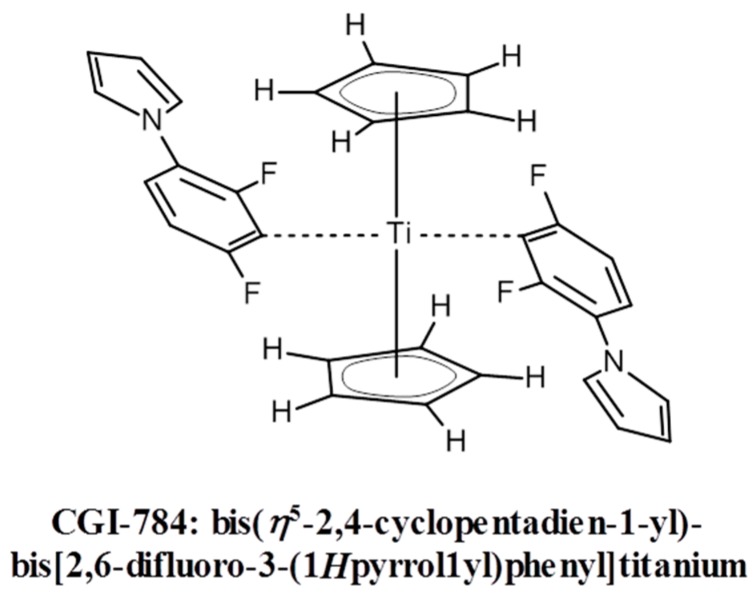
Structure formula of the titanocene complex CGI-784.

In this system, irradiation of the photo initiator (*PI*) provides the reactive species for the initiation step. In more detail the *PI* absorbs the light energy and transforms intra molecularly into a reactive species, here an initiating radical *R*^•^ that attacks a monomer *M* and finally converts into the (photo) polymer (Scheme 1):

Titanocene derivatives represent one of the few examples of photo-initiators for the visible spectral range (VIS) that are directly photolyzed upon light exposure. More examples can be found in [36]. The use of titanocene complexes (e.g., CGI-784 from BASF AG, Ludwigshafen, Germany, see Scheme 2) in experiments where several high refractive index organic monomers were incorporated into acrylate oligomer-based formulations, irradiated at 546 nm was described in [37].

The presence or absence of oxygen (in *N*_2_) plays a vital role in the initiation process as was shown by studies with different concentrations of titanocene and at different incident light intensity. In aerobic and anaerobic atmospheres it was observed that the polymerization reaction commenced rapidly. After reacting for a few seconds, this process however suffered abrupt deactivation, being more pronounced at low titanocene concentration [38]. Additionally, photo bleaching of the titanocene requires rather harsh conditions like high UV dosages which lead to some residual coloration in the recorded material. The addition of well-known additives into the photo initiating system (i.e., diphenyliodonium salt, *N*-methyldiethanolamine, etc.) raises the efficiency of the photo initiation [39]. These additives utilize the photo chemically generated radicals that may inhibit the polymerization process to form new radicals active in polymerization. As these additives now play a vital role in the photo initiating system they transform it virtually into a two component photo initiator.

### 4.2. Two-Component System Dye/Organo-Borate Salt

Two-component photo initiating systems are the commonly used material combination that allow for an efficient photo-polymerization in the VIS. They contain one component to absorb the light for example a sensitizer dye and one component that interact with the sensitizer dye to form the initiating species. In general they follow a bimolecular and a stepwise process consisting of a first step of light absorption by the photo-initiator or dye (i.e., thioxanthones, ketocoumarines, etc.) and a second step by atom or electron transfer through a co-initiator (i.e., alcohols or thiols serve as hydrogen donors and/or amines serve as electron donors) that subsequently generates the initiating species. Especially for electron transfers photo-initiators an additional way of deactivation has to be considered—the back electron transfer.

**Scheme 3:**
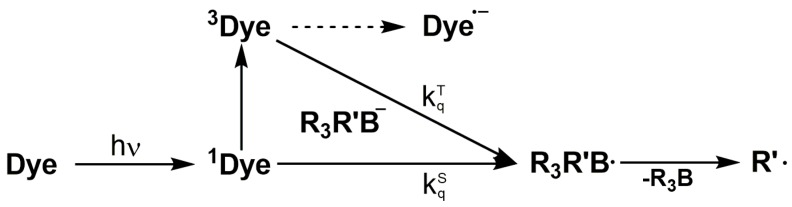
Reaction of a two-component photo-initiator system.

Combinations of selected dyes with triarylalkyl-borate salts have been reported as co-initiators for Norrish type II photo-initiating systems [40]. The initiation mechanism of the radical chain formation involves a photo-induced electron transfer from the borate salt to the singlet excited state (^1^Dye) or triplet state (^3^Dye) of the dye [41]. Subsequently, the boranyl radical R_3_R’B^•^ is formed, which may undergo a carbon-boron bond dissociation generating an alkyl radical (Scheme 3). The rate constant for the mentioned electron transfer reaction correlates with the Gibbs free energy change (Δ*G_et_*). Furthermore, the rate of the C–B cleavage depends on the thermodynamic stability of the primary radical formed. For example the lifetime of the triphenyl-butyl boranyl radical is reported to be the range of 250 fs [42], preventing any non-productive back electron transfer reaction. A potential dependency of the polymerization efficiency on the structure of the borate salt is still contradictory discussed, since studies exist with a correlation while other studies reveal that a specific behavior is not fully understood [43,44].

**Scheme 4:**
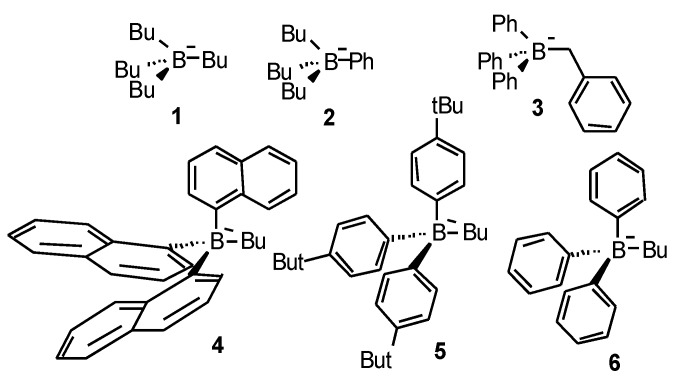
Borate anions used in this study (counter ions are ^+^NMe_4_ or ^+^NBu_4_).

A good way to investigate and characterize the photo chemistry of these type (II) photo initiators is to study the decay of an acrylic reactive thinner that readily dissolves the photo initiator by online IR spectroscopy. The obtained kinetic data allow us to deduce potential reaction mechanism, relative reactivities and chemical stabilities. Rates of conversion *R_p_* and final conversion *C* obtained from the photo-polymerization experiments of an acrylate resin under laser irradiation at 532 nm are collected in Table 1, using the borate salts given in Scheme 4.

**Figure 6:**
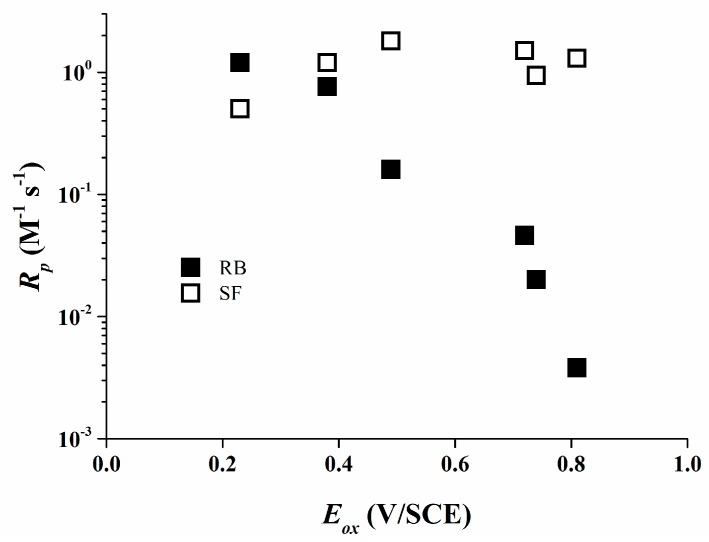
Rate of polymerization *R_p_* for Rose Bengal (**RB**) (filled symbols) and Safranine O (**SF**) (open symbols) versus the oxidation potential *E_ox_* of the borate salts [35].

The high reactivity of borate salts as co-initiators is demonstrated due to their high final conversions for most of the systems used. For the Rose Bengal/organo-borate systems *R_p_* values decrease continuously with increasing *E_ox_* (Figure 6). The maximum *R_p_* was detected for **1** which exhibits the least *E_ox_*. An about 300 times reduced *R_p_* value was determined for **6** which exhibits a higher *E_ox_* than that of **1**.

This is consistent with the assumption that the efficiency of the photo-polymerization reaction depends mainly on the primary electron transfer process. In the case of Safranine O/organo-borate salts, *R_p_* does not correlate with *E_ox_* of the borates (Figure 6) and *R_p_* varies only by a factor of 4.

The reactivity is very high for all the organo-borates, quite close to the highest *R_p_* found for Rose Bengal/organo-borate complexes. Thus, Rose Bengal and Safranine O significantly vary in their reactivity as a part of a type II photo-initiating system.

### 4.3. Excited State Process

The Gibbs free energy ∆*G_et_* accompanying the electron transfer reaction appearing within the dye/organo-borate combinations can be estimated using the Rehm-Weller equation [45]

(6) $$\begin{array}{l} \Delta G_{et}^{S}=E_{ox}-E_{red}-E_{S}\\ \Delta G_{et}^{T}=E_{ox}-E_{red}-E_{T} \end{array}$$

With ∆*G_et_^S^* and ∆*G_et_^T^* defined as Gibbs free energy for the electron transfer to the excited singlet state and excited triplet state, respectively. *E_red_* is defined as reduction potential of the dye, *E_S_* and *E_T_* for the singlet or triplet energy of the dye. The Rehm-Weller equation neglects the coulomb term. The corresponding values for all possible dye/organo-borate combinations are thermodynamically favorable. Therefore it deserves a more detailed mechanistic study to understand the excited state’s reactivity.

Fluorescence spectroscopy was used to study the singlet excited states of the dyes and laser flash photolysis (LFP) for their triplet excited states. The corresponding excited state quenching rate constants (*k_q_^S^* or *k_q_^T^*) are accessed from the Stern-Volmer analysis of the decay curves

(7) $$\frac{1}{\tau }=\frac{1}{\tau_{0}}+k_{q}^{S,T}\cdot \left[Q\right]$$

τ and τ_0_ are the lifetimes of the excited states with or without the quencher *Q*—the organo-borate salt in this case.

The rate constant *k_q_^S^* of the reaction of the singlet excited states ^1^**RB** and ^1^**SF** with the organo-borate salts was detected using fluorescence spectroscopy with excitation at 550 nm for **RB** and 460 nm for SF (Table 2). ^1^**SF** leads to more or less diffusion controlled reactivity in acetonitrile as solvent, which is in agreement with the rather negative values for ∆*G_et_^S^*. In the opposite case of ^1^**RB**, although the ∆*G_et_^S^* values are rather low, much more reduced *k_q_^S^* values (below the diffusion limit) were observed.

Laser flash photolysis studies were done at 532 nm in acetonitrile for both **RB** and **SF**. The lifetime of ^3^SF decreases when adding the organo-borate salt which was monitored at 830 nm. The associated quenching rate constants *k_q_^T^* are diffusion controlled for the most negative values of ∆*G_et_^T^* (i.e., for **4**) and are diminished by a factor of 20 for **6** (Table 2). The ground state photo-bleaching of Safranine O at relatively long time scales, which was monitored at 510 nm, displays a higher population with increasing concentration of organo-borate salt. This can be interpreted as a consequence of the formation of the semi-reduced form of Safranine O. The latter transient species was detected at 430 nm and displays an increase with increasing concentration of borate salt. This is the result of the reactivity of both the singlet and triplet excited states of Safranine O with the organo-borate salts.

The triplet state of Rose Bengal can be monitored by LFP at 610 nm in acetonitrile. The observed data for the different triplet state quenching rate constants are collected in Table 2. The *k_q_^T^* values behave inversely proportional with ∆*G_et_^T^*. More interestingly, even with such a diminished value as −0.57 eV for ∆*G_et_^T^* a low quenching rate constant of 2.2 × 10^6^ M^−1^∙s^−1^ is observed, i.e., a ~300 times reduced reactivity than for Safranine O at similar ∆*G_et_^T^* (see ^3^**SF**/**4**, *k_q_^T^* = 6.8 × 10^9^ M^−1^∙s^−1^).

Under excitation by light Rose Bengal therefore reacts with the organo-borate salts from both the singlet and the triplet state. This results in a ground state photo-bleaching of Rose Bengal which can be detected at 550 nm. Concomitantly, the Rose Bengal semi-reduced form, which is generated during this reaction, was detected at 430 nm in acetonitrile, proving the electron transfer. The obtained quenching rate constants of the singlet and the triplet excited states of Rose Bengal increase with the decreasing of the oxidation potential of organo-borate salts. These results are in line with the measured photo-initiating efficiency of the respective reaction partners (*cf.* Table 1).

**Figure 7:**
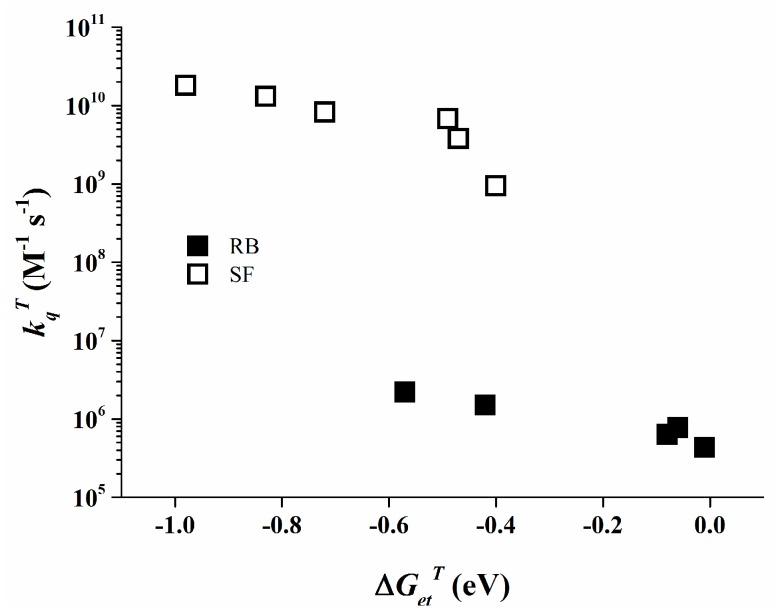
Quenching rate constant *k_q_^T^* of **RB** (filled symbols) and of **SF** (open symbols) as a function of ∆*G_et_^T^* for different borate salts [35].

The data as shown in Table 2 clearly show that Safranine O undergoes a diffusion controlled electron transfer process when ∆*G_et_* is significantly negative, a known behavior for bimolecular photo-induced electron transfer [46]. The comparable behavior of Rose Bengal is less conventional since Rose Bengal exhibits rather low quenching rate constants even at significantly negative ∆*G_et_* values, as depicted in Figure 7 for its triplet state. This specific behavior of Rose Bengal can be attributed to relatively low electronic coupling between the Rose Bengal excited states and the organo-borate salt [47,48].

It has been widely reported in the literature that the efficiency of the photo-polymerization process is directly proportional to the excited state reactivity and that a good mechanistic understanding of this reactivity in solution allows the stringent interpretation of the polymerization data. These experiments, performed in low viscous solutions, however, rarely take into account the specific features of such photo-polymerizing resin. The rate constants of the monitored reaction in solution have to be corrected for the diffusion of the monomer in the medium by taking into account the viscosity η of the respective resin and calculating the diffusion rate constant from a simplified Stokes-Einstein equation:

(8) $$k_{d}=\frac{8\cdot R\cdot T}{3\cdot \eta }$$

The value of the material described herein is *k_d_* = 4.2 × 10^6^ M^−1^∙s^−1^. The current quenching rate constants *k_r_* under such conditions can be evaluated from Equation (9):

(9) $$k_{r}=\frac{k_{d}\cdot k_{q}}{k_{-d}+k_{q}}$$

With *k*_−*d*_ expressing the separation rate constant in the medium which was set equal to *k_d_* [45,46,47,48,49,50]. As a consequence, the rate constant of the reaction between the organo-borate salts and excited dyes will be limited by diffusion for the Safranine O/organo-borate systems. In the case of the Rose Bengal/organo-borate systems, the quenching rates constant are limited by diffusion for the singlet state but still below the diffusion limit for the triplet state.

Apart from the correction for the diffusion in the described medium, it has to be taken into account that the quantum yield for the formation of the alkyl radical φ*_Ρ_* additionally depends on the photo-physical properties of the excited states, the quenching rate constants *k_r_* and the concentration of quencher [*Q*]. φ*_R_* can be thus expressed as the sum of the contributions from both the singlet state φ*_Ρ_^S^* and the triplet state φ*_R_^T^*, respectively. φ*_R_^S^* only depends on the singlet state quenching efficiency of the dye by the organo-borate as:

(10) $$\varphi_{R}^{S}=1-\frac{1}{1+k_{r}^{S}\cdot \tau_{0}^{S}\cdot \left[Q\right]}$$

where τ_0_*^S^* expresses the lifetime of the singlet excited state of the dye in the absence of a quencher. Similarly, φ*_R_^T^* depends on the triplet state quenching efficiency and the quantum yield of the actual triplet state φ*_T_.*

(11) $$\varphi_{R}^{T}=\varphi_{T}\cdot \left(1-\frac{1}{1+k_{r}^{T}\cdot \tau_{0}^{T}\cdot \left[Q\right]}\right)$$

With τ_0_*^T^* as the lifetime of the actual triplet excited state. φ*_T_* moreover is given by:

(12) $$\varphi_{T}=\frac{\varphi_{T}^{0}}{1+k_{r}^{S}\cdot \tau_{0}^{S}\cdot \left[Q\right]}$$

φ*_T_*^0^ being the triplet state quantum yield in the absence of a quencher. Therefore, the overall quantum yield φ*_R_* of alkyl radical formation is expressed by:

(13) $$\varphi_{R}=\left(1-\frac{1}{1+k_{r}^{S}\cdot \tau_{0}^{S}\cdot [Q]}\right)+\left(\frac{\varphi_{T}}{1+k_{r}^{S}\cdot \tau_{0}^{S}\cdot [Q]}\right)\left(1-\frac{1}{1+k_{r}^{T}\cdot \tau_{0}^{T}\cdot [Q]}\right)$$

The values of φ*_R_* are shown in Table 3. It can be deduced from these data that the total quantum yield of the product formation φ*_R_* for the photo-initiating systems based on Rose Bengal/organo-borate salt increases with *E_ox_* as a consequence of the rather low values of the quenching rate constants. In case of Safranine O, the φ*_R_* values are similar to each independent of the organo-borate salt used, since the quenching rate constants *k_r_* in the resin are diffusion limited.

**Figure 8:**
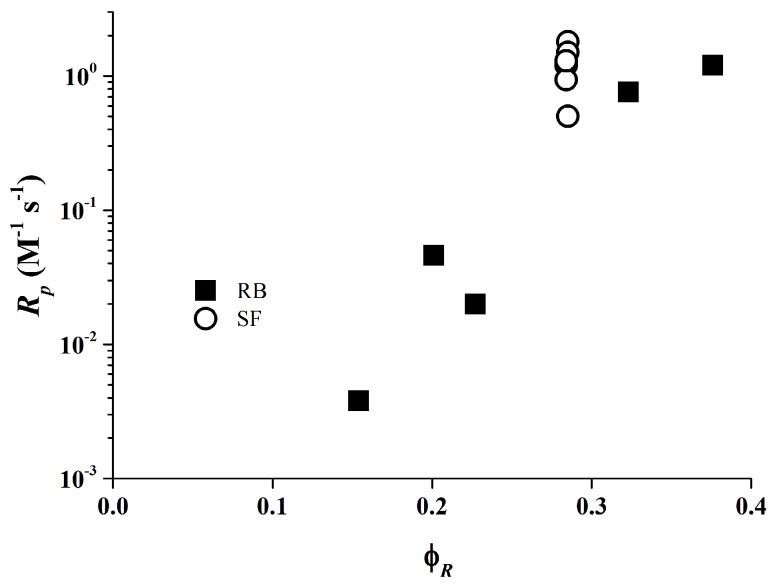
*R_p_* versus φ*_R_* in the photo-polymerizing resin [35].

Figure 8 exhibit that the rate of polymerization for both Rose Bengal and Safranine O fit a relationship with φ*_R_*. These data are consistent with the results obtained from the RT-FTIR experiments where the photo-initiating system consisting of Rose Bengal/organo-borate **4** is the most efficient system. From these data, the efficiency of the systems based on Safranine O/organo-borate salts is in the diffusion limit and their reactivity is not proportional to the photo-induced electron transfer process.

### 4.4. Holographic Recording

**Figure 9:**
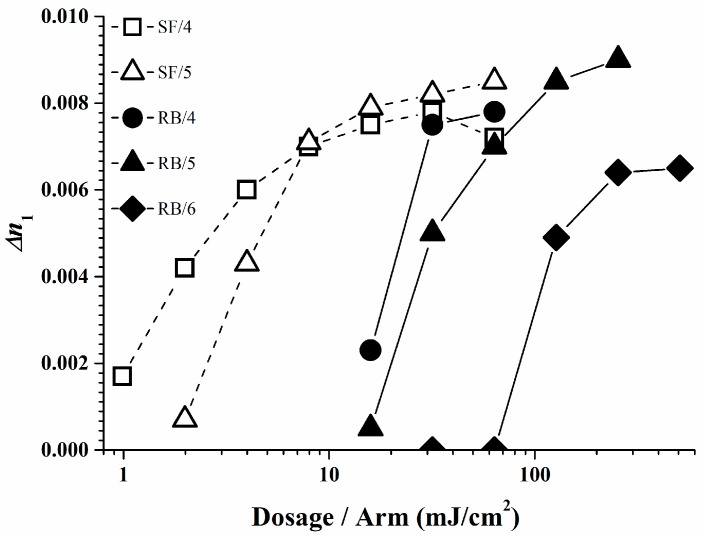
∆*n*_1_ as a function of the irradiation dosage per arm [35].

The photopolymer formulations of Rose Bengal and Safranine O with respective borates were investigated in a holographic experiment. Unfortunately not all the dye/organo-borate combinations form stable photo-initiators due to their tendency to undergo a spontaneous self- or dark reaction. For the suitable photo-initiators holographic plane wave grating recordings were performed using the dye/organo-borate photo-initiating system in a photopolymer composed of 25% SR 349, 0.1% dye, and 1.0% organo-borate in a matrix [13,18]. Figure 9 and Table 3 display the refractive index modulation ∆*n*_1_ of the holograms obtained for stable photopolymers as a function of the irradiation dose per arm of the 2-beam plane wave recording setup. Note high ∆*n*_1_ values indicate high brightness upon vHOE reconstruction, provided over-modulation has not occurred or cannot occur as for reflection holograms. Recording was done in reflection mode with green laser light of 532 nm. Experimental details of the recording setup and data extraction are described elsewhere [17]. Good ∆*n*_1_ values and high sensitivity were found for the systems based on Safranine O/organo-borate **5** and Safranine O/organo-borate **4**. For the Rose Bengal based systems, also good ∆*n*_1_ values and medium sensitivity was found when using organo-borates **4** and **5**, good ∆*n*_1_ values and poor sensitivity was found for organo-borate **6**.

**Figure 10:**
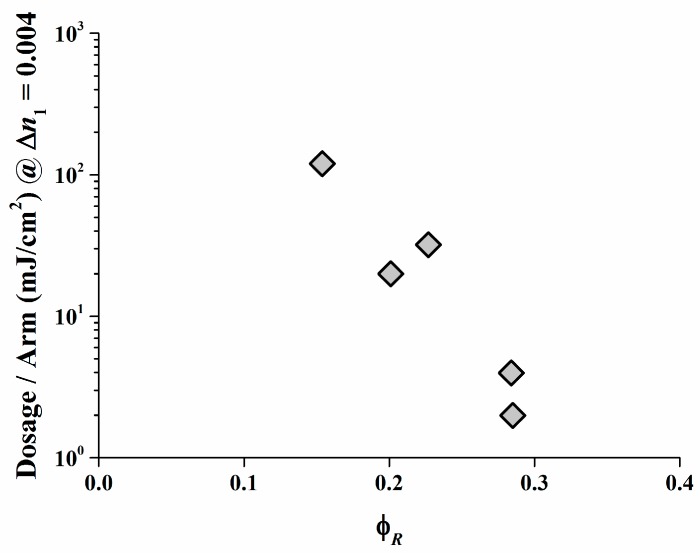
Dosage per arm necessary to obtain a ∆*n*_1_ = 0.004 as a function of φ*_R_* [35].

As displayed, a low irradiation dose is required only to create a high ∆*n*_1_ in the Safranine O/organo-borate **4** and Safranine O/organo-borate **5** systems as a result of the high radical quantum yield φ*_R_* created and the high *R_p_* values. A high ∆*n*_1_ is also obtained for Rose Bengal/organo-borate **4** and Rose Bengal/organo-borate **5**, although in this case, a longer exposure time is necessary due to the lower *R_p_* value. Finally, for the Rose Bengal/organo-borate **6** system, a moderate ∆*n*_1_ is obtained at high exposure dosage, due to the very low *R_p_* and low radical quantum yield φ*_R_*. Surprisingly, the sequence of increasing inhibition dosages (the dosage needed to start any holographic recording) for each dye exactly follows the sequence of decreasing *R_p_* values for each dye/organo-borate couple. By taking e.g., a value of ∆*n*_1_ = 0.004, which corresponds to half of the maximum value obtained in holographic recording, Figure 10 correlates the corresponding dosage per arm with φ*_R_* in a viscous medium. Thus, the results of the holographic recording as described quite well correlate with the photo-polymerization experiments.

The ability to predict and interpret the performance of a photo reactive polymer resin containing a type II photo-initiating system from the reactivity of the excited states of the involved dyes and from the intrinsic properties of the photo-polymerizing medium has been demonstrated.

## 5. Full Non-Local Reaction Driven Diffusion Model for Bayfol^®^ HX Film

We have shown in the previous Section 3 that the recording process in Bayfol^®^ HX film can be successfully described by a simple non-local reaction diffusion model. Having investigated in Section 4 the details of the photo-initiation mechanism we are now able to extend our simple non-local reaction diffusion model to a fully detailed model. By this we are able to eliminate many simplifications and shortcomings of the earlier model. In [51] we have shown how the detailed photo-initiation process, the chain growth and termination processes, the inhibition effects due to oxygen and the mass transport of all relevant mobile species including the counter flow of a non-polymerizable component, to guarantee incompressibility can be incorporated. We will firstly review this work given in [51] to some extend to rationalize later the photopolymer formulation optimization for a tunable index modulation ∆*n*_1_ and mechanical stiffness (measured as storage shear modulus *G_photo cured_*).

### 5.1. Outline of the Fully Detailed Model

As outlined in [51] the description of the photo-initiation process is the starting point. First a photon is absorbed by the dye *A*. *A* will then be converted to its excited triplet state Dye* with a rate constant *k_a_*. The dye *A* can be recovered from its excited triplet state Dye* with a decay constant *k_r_*. *k_r_* is the inverse of the lifetime τ*_r_* of the excited triplet state Dye*.

(14) $$A\overset{\;k_{a}\;}{⟶}Dye*$$

(15) $$Dye*\overset{\;k_{r}\;}{⟶}A$$

For the formation of the primary radical molecule *R*^•^ the excited triplet state Dye* reacts with a co-initiator *CI* with a rate constant *k_d_*. Besides *R*^•^ a protonated dye molecule *HDye* is formed too.

(16) $$Dye*+CI\overset{\;k_{d}\;}{⟶}Dye^{•-}+R^{•}+H^{+}\overset{\;}{⟶}HDye+R^{•}$$

A finally bleached dye is generated by the reaction of the protonated dye molecule *HDye* with the co-initiator *CI*. An intermediate co-initiator state *CI*_int_ is also formed.

(17) $$HDye+R^{•}\overset{\;k_{b}\;}{⟶}\mathrm{bleached}\;\mathrm{dye}+CI_{\mathrm{int}}$$

For the initiation of a chain initiator molecule *M*_1_^•^ the primary radicals *R*^•^ react with a monomer *u* with a rate constant *k_i_*. There may not be only a single type of monomer present but a mixture of monomers with for example different functionalities could be used. Monomers with higher functionality will form an interpenetrating network with the matrix network. Different monomer types are not further distinguished from the chain initiator state on. Also all mobility of the resulting chain and network molecules is assumed to be lost from this state on within our model.

(18) $$u+R^{•}\overset{\;k_{i}\;}{⟶}M_{1}^{•}$$

For monomers with functionalities *f* > 1 it might be reasonable to assume that the initiation rate constant *k_i_* scales with *f*.

After the initiation process the chain propagation has to be described by the rate constant *k_p_*.

(19) $$u+M_{n}^{•}\overset{\;k_{p}\;}{⟶}M_{n+1}^{•}$$

The driving force for monomer diffusion is generated by the assumed local immobilization of the polymerized monomers. Therefore it might be reasonable to assume that the propagation rate constant *k_p_* is independent of the functionality of the monomer type. If several types of monomers are used they should have the same chemical type of functional group (e.g., an acrylate group) to keep the latter assumption valid.

After chain growth chain termination has to be described. Combination or disproportionation is a consequence of bimolecular termination as depicted below.

(20) $$M_{m}^{•}+M_{n}^{•}\overset{\;k_{tc}\;}{⟶}M_{m+n}$$

(21) $$M_{m}^{•}+M_{n}^{•}\overset{\;k_{td}\;}{⟶}M_{m}+M_{n}$$

It is common to combine the two respective rate constants in one bimolecular termination rate constant *k_t_*:

(22) $$k_{t}=k_{tc}+k_{td}$$

A terminated polymer is also achieved by the termination of a growing chain radical with a primary radical.

(23) $$R^{•}+M_{n}^{•}\overset{\;k_{tp}\;}{⟶}RM_{n}$$

Primary radical recombination will be neglected in this study. For high recording powers especially in the case of mono pulse recording (~ns pulse time) this assumption has to be revised again.

Besides initiation, growth and termination the inhibition as a non-ideal effect has to be included. For free radical polymerization oxygen (in the following denoted as *Z*) usually governs inhibition. Oxygene (*Z*) is able to react with the excited triplet state of the dye, the primary radical or the macro radical.

(24) $$Dye*+Z\overset{\;k_{z1}\;}{⟶}\mathrm{oxydized}\;\mathrm{leuco}\;\mathrm{dye}$$

(25) $$R^{•}+Z\overset{\;k_{z2}\;}{⟶}\mathrm{scavengend}\;\mathrm{radical}$$

(26) $$M^{•}+Z\overset{\;k_{z3}\;}{⟶}\mathrm{terminated}\;\mathrm{polymer}$$

The following assumptions can be made considering the mass transport of the different mobile species in the photopolymer. *Z* may diffuse from the plastic substrate into the photopolymer at a rate proportional to–(*Z–Z*_0_). *Z*_0_ is denoted as the equilibrium concentration of oxygen in the photopolymer layer. Within the photopolymer layer *Z* diffuses too. Mobile components are considered to be monomers *u*, the primary radical *R*^•^ and all other components of the photo-initiator system. Within this model we consider as immobile components all chain radicals (they may be also cross linked in case of monomer functionalities *f* > 1) and the cross linked matrix. A non-reactive contrast agent *B* is introduced to compensate the mass flow of monomers from the dark fringe towards the bright fringe during the holographic recording and by this equalizing the osmotic pressure. By the counter-diffusion of the contrast agent *B* to the monomers *u* the incompressibility is given at any time at any location. The contributions to incompressibility violation by the initiator system components are neglected as they are only present in very dilute concentrations. Still neglected for incompressibility violation is volume shrinkage that occur for example in free radical polymerization of acrylic monomers. In Bayfol^®^ HX film photopolymers for example the volume shrinkage is below 3%, depending on the composition.

In the presence of a spatially sinusoidal variation of the intensity during recording of a plane-wave grating the reactions described above have to be translated into a set of integro-differential equations. In order to solve the set of integro-differential equations it is straightforward to expand them into a Fourier series of the grating period Λ of the respective grating vector length *K* = 2π/Λ.

In [51] we further outlined that the rate constant *k_a_* that describes the excitation rate of the dye *A* to its excited triplet state Dye^*^ is expressed as:

(27) $$k_{a}=\Phi \cdot \varepsilon \cdot d\cdot I_{0}^{\prime }$$

(28) $$\varepsilon =\frac{\ln\left(T_{sf}/T_{0}\right)}{A_{0}\left(t=0\right)\cdot d}$$

(29) $$I_{0}^{\prime }=\frac{T_{sf}\cdot I_{0}\cdot \lambda }{N_{A}\cdot h\cdot c}\cdot \frac{1-\exp\left[-\varepsilon \cdot A_{0}\left(t\right)\cdot d\right]}{d}\cdot \Theta \left(t\right)\cdot \Theta \left(t_{\exp}-t\right)$$

where *d* is the traversed thickness of the light according to the recording conditions given in cm. ε is the molar extinction coefficient given in cm^2^/mol. Φ being identical to φ*_T_*^0^ from Section 4 is the quantum efficiency to generate an excited triplet state Dye^*^ per photon that traverses the photopolymer. *T*_0_ is the transmission at the recording wavelength before illumination and *T_sf_* is the final transmission after recording and is set to 0.9. *A*_0_ is the initial zero-order spatial Fourier component of the dye concentration, given in mol/cm^3^. *N_A_* is the Avogadro’s constant, *h* the Planck’s constant and *c* the vacuum speed of light. Θ denotes the Heaviside function. The average number of absorbed mol photons per second and per unit volume through the photopolymer layer during the full recording interval (0, *t*_exp_) is described by Equation (29). *A*_0_(*t*) denotes the transient zero order Fourier component of the dye distribution. By *A*_0_(*t*) also the transient bleaching during the recording interval (0, *t*_exp_) is taken into account within this model.

Rate equations for the extensive variables were formulated and the details are given in [51]. One example is the rate equation for the dye *A*. All concentrations are given in mol/cm^3^.

(30) $$\frac{\partial A}{\partial t}=\frac{\partial }{\partial x}\left[D_{A}\frac{\partial A}{\partial x}\right]-k_{a}\cdot \left(1+V\cdot \cos\left(K\cdot x\right)\right)\cdot A+k_{r}\cdot Dye*$$

Another example is the rate equation for the monomer *u*.

(31) $$\frac{\partial u}{\partial t}=\frac{\partial }{\partial x}\left[D_{u}\frac{\partial u}{\partial x}\right]-k_{i}\cdot R^{•}\cdot u-\int_{-\infty }^{\infty }\frac{1}{\sqrt{2\pi \cdot \sigma^{2}}}\cdot \exp\left[-\frac{{\left(x-x\prime \right)}^{2}}{2\cdot \sigma^{2}}\right]\cdot k_{p}\cdot M^{•}\cdot u\cdot dx\prime$$

The non-local parameter is denoted as σ^2^ again [22]. As reported in [17], σ^2^ was set to 100 nm^2^.

For the polymerized monomer units *N* the following rate equation can be formulated:

(32) $$\frac{\partial N}{\partial t}=\int_{-\infty }^{\infty }\frac{1}{\sqrt{2\pi \cdot \sigma^{2}}}\cdot \exp\left[-\frac{{\left(x-x\prime \right)}^{2}}{2\cdot \sigma^{2}}\right]\cdot k_{p}\cdot M^{•}\cdot u\cdot dx\prime$$

The rate equation for the non-polymerizable contrast agent *B* has to account for the incompressibility and as a consequence has to be written as:

(33) $$\frac{\partial B}{\partial t}=-VF\cdot \frac{\partial }{\partial x}\left[D_{u}\frac{\partial u}{\partial x}\right]$$

The ratio of molar volumes of the monomer *u* and the contrast agent *B* is denoted as *VF*.

The zero-order Fourier component of the polymerized monomer units *N*_0_, which is a measure of the spatially averaged monomer conversion and by this a measure for the increasing internal viscosity, was used in [51] to describe the slowdown of diffusion speed of all mobile components. Therefore the diffusion coefficients *D* are scaled down linearly in *N*_0_(*t*) via:

(34) $$D=D_{0}-\left(1-\frac{1}{\alpha }\right)\cdot D_{0}\cdot \frac{N_{0}\left(t\right)}{u_{0}\left(t=0\right)}$$

From rheological properties like the ratio of the shear modulus of the unrecorded state *G*_0_ versus the shear modulus in the fully cured state *G_photo cured_* the scaling constant α can be deduced. Due to the possible large concentrations and concentration gradients of the monomers *D_u_* has the character of an inter-diffusion coefficient. Due to small concentrations of the initiator system components in the photopolymer formulation *D_A_*, *D_HDye_*, *D_CI_* and *D_R_*^●^ have the character of tracer diffusion coefficients. 

Further we relate the diffusion coefficients *D*_0_ of the various mobile components to that of the monomer unit in a rubbery network (Equation (29) in [51]) by the following scaling:

(35) D0∝(ρM)43

For further simplification we set *D_A_* = *D_HDye_* and *D_CI_* = *D_R_*^●^.

By Fourier-series expansion with respect to the basic grating period Λ the above mentioned set of integro-differential equations under appropriate initial conditions can be solved.

**Figure 11:**
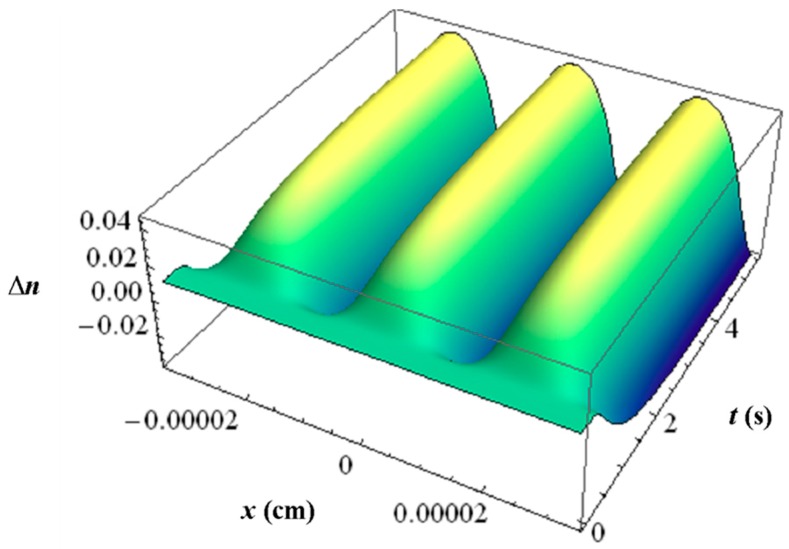
Simulated refractive index profile ∆*n* versus time *t* and space coordinate *x* for Bayfol^®^ HX film, calculated for λ = 633 nm, *SF* = 4525 1/mm, *I*_0_ = 9.12 mW/cm^2^ and *t*_exp_ = 3 s [51].

With all the respective volume fraction profiles φ*^k^* of the monomers *u*, the polymerized monomers *N*, the contrast agent *B* and the matrix *b* as background and their different refractive indices, the refractive index profile ∆*n* can be calculated using the Lorentz-Lorenz relation and the incompressibility condition. In Figure 11 an example of such a ∆*n* profile is depicted with the inhibition period clearly visible.

In the two wave approximation outlined in Kogelnik’s work [1] for the first diffraction order of the hologram only the first order Fourier components ∆*n*_1_ of the refractive index profile ∆*n* is of interest. At each time *t* we can therefore write for ∆*n*_1_, which is then identical to the index modulation of the holographic grating:

(36) $$\begin{array}{l} \Delta n_{1}=\frac{{\left(n_{dark}^{2}+2\right)}^{2}}{6\cdot n_{dark}}\cdot \\ \left[\varphi_{1}^{u}\cdot \left(\frac{n_{u}^{2}-1}{n_{u}^{2}+2}-\frac{n_{b}^{2}-1}{n_{b}^{2}+2}\right)+\varphi_{1}^{B}\cdot \left(\frac{n_{B}^{2}-1}{n_{B}^{2}+2}-\frac{n_{b}^{2}-1}{n_{b}^{2}+2}\right)+\varphi_{1}^{p}\cdot \left(\frac{n_{p}^{2}-1}{n_{p}^{2}+2}-\frac{n_{b}^{2}-1}{n_{b}^{2}+2}\right)\right] \end{array}$$

Here *n_dark_* denotes the refractive index of the unrecorded photopolymer, *n_u_* the refractive index of the monomer, *n_B_* the refractive index of the contrast agent, *n_N_* the refractive index of the polymerized monomers and *n_b_* the refractive index of the matrix. These refractive indices were measured independently in our case. φ_1_*^k^* is the first-order Fourier component of the volume-fraction profile φ*^k^* of the component *k* of the photopolymer. Extension to a multi-component monomer mixture is straightforward.

### 5.2. Estimation of the Model Parameters by Comparing Experimental and Simulated Dosage Response Curves of RGB Sensitive Bayfol^®^ HX Film

**Figure 12:**
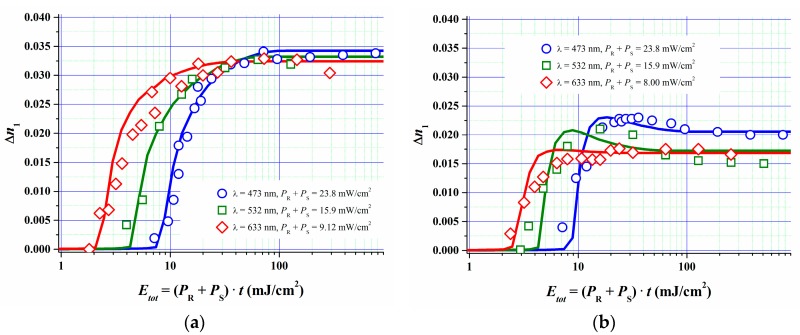
Experimental and simulated dosage-response curves for RGB sensitive Bayfol^®^ HX film; (**a**) reflection geometry data; (**b**) transmission geometry data [51].

In [51] we described how we estimated the parameters for the model described above by fitting the model to the measured R, G and B dosage-response curves of a specific Bayfol^®^ HX film grade at two different spatial frequencies. Independently measured refractive indices of all relevant components were used. Also experimental data for *k_p_* [52] and for *k_d_* and *k_r_* for the formulation components were set as starting values for the fit procedure. *k_d_* and *k_r_* were deduced from respective experiments as described in Section 4. For example the lifetime τ*_r_* of the excited triplet state of the dye was found to be of the order of 10 µs, therefore *k_r_* is of the order of 1/10 µs^−1^. According to Section 4 one can identify *k_q_* = *k_d_*∙*CI*_0_ (*t* = 0) from the experimentally accessible quenching constant *k_q_* of the excited triplet state Dye^*^. As the whole set of rate equations can be scaled by an arbitrary factor in time it is important to know at least a few of the above mentioned rate constants. This then leads to a consistent set of physically reasonable model parameters. In Figure 12 the obtained results are shown.

After parameter value optimization the simulations show a very good fit quality to the experiments. The good fit quality is underlined by the fact that the parameters for the polymerization (*k_i_*, *k_p_*, *k_t_*, *k_tp_*, *k_z_*_2_, *k_z_*_3_, σ^2^) and mass transport of monomer and oxygen (*D_u_*, α, *D_Z_*, *Z*_0_, τ [51]) were kept almost identical in all 6 cases (3 different λ and 2 different *SF*) depicted in Figure 12. In addition parameters related to the initiator system (φ, *k_r_*, *k_d_*, *k_b_*, *D_A_*, *D_CI_*) could be kept fixed for the different *SF* for reflection and transmission geometry for each recording wavelength. Only for the blue recording in transmission geometry φ, *k_r_*, *k_d_*, *k_b_* and *k_i_* had to be chosen significantly different from the other cases (~50%).

In sum we can state that the fully detailed non-local reaction-diffusion model is able to describe our photopolymer recording behavior very well in a wide variety of recording conditions and also in wide variations of spatial frequencies.

### 5.3. Improving the Spatial Frequency Response Based on the Fully Detailed Reaction-Diffusion Model

As we reported previously in Section 3, as a consequence of the low spatial frequency cutoff, ∆*n*_1_ for transmission holograms in saturation recording is only about half the value of ∆*n*_1_ for reflection holograms at comparable power densities. This fact can be easily rationalized by the picture that at large grating spacing Λ (low spatial frequencies *SF*) the diffusing monomers already react on their travel to the bright fringe before reaching the bright fringe’s center. By that higher harmonic orders in the refractive index profile ∆*n*(*x*, *t*) are built up. Therefore only part of the complete dynamic range of the photopolymer is transformed into the desired first Fourier component ∆*n*_1_. From the model predictions we can expect that increasing the monomer mobility, by changing the composition of photopolymer formulation should improve the ∆*n*_1_ at large grating spacing.

The monomers can be selected as a mixture of monofunctional and multifunctional species. Upon photo-polymerization the multifunctional species together with the monofunctional species generate a rigid cross linked polymer network. Multifunctional monomers will generate a very rigid network that gives the hologram long term stability. A too high amount of multifunctional species however vitrifies this photopolymer network already at low monomer conversion and decreases monomer mobility at an early stage. On the other hand a high amount of small, monofunctional monomers generates flexible linear segments in the network and allows the monomer mobility to be kept high even at elevated monomer conversion.

Different photopolymer formulations were evaluated in red laser (633 nm) reflection hologram recording to test this assumption [51]. The total weight fraction of a monomer mixture that consists of a mono- and a multifunctional monomer species was kept constant in these formulations. To assure that any effect on ∆*n*_1_ caused by varying the monomer mixing ratio can be attributed to the change in monomer mobility, the monofunctional and the multifunctional monomers were chosen such that they have almost the same double-bond density per volume (meaning also that the molecular weight of the monofunctional monomer is proportionately smaller than that of the multifunctional monomer), the same chemical type of double bond (acrylate) and almost identical refractive indices.

**Figure 13:**
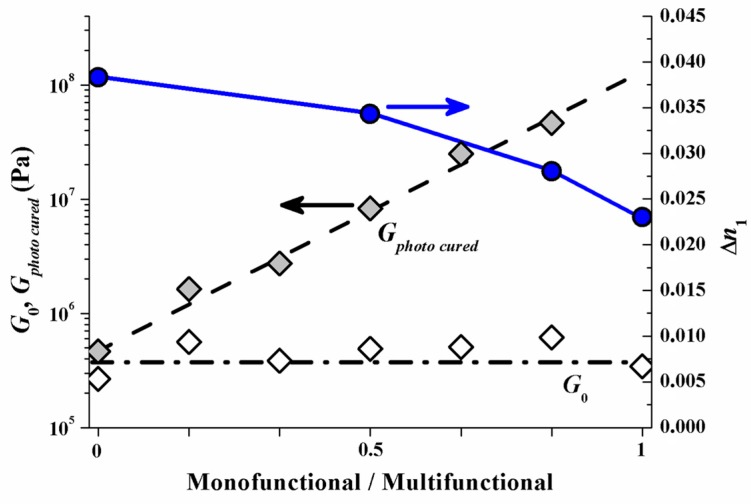
∆*n*_1_ (**right** *y*-axis, blue circles) and shear modulus *G* (**left** *y*-axis), before (open squares) and after flood cure (grey squares), versus monomer mixing ratio (fraction of the multifunctional monomer increasing from 0 to 1) [51].

As a result of this experimental test the index modulation ∆*n*_1_ versus the monomer mixing ratio is depicted (right *y*-axis) in Figure 13. In addition to represent the mechanical rigidity the shear modulus *G*, measured with an optical Rheometer (Physica MCR 301, method describe in [53]) is shown for the unrecorded state as *G*_0_ and for the homogeneously photo cured state as *G_photo cured_* (left *y*-axis). It is very obvious that if the multifunctional monomers are the majority component ∆*n*_1_ drops significantly. This drop in ∆*n*_1_ by increasing amount of multifunctional monomers can be attributed to the early densification of the photopolymer network and therefore strong attenuation of the monomer diffusion constant. Note that *G*_0_ does not change with the monomer mixing ratio. This suggests identical rheological conditions for the monomers to diffuse in the very initial state of exposure.

Reflection hologram recording shows a high spatial frequency *SF*. Therefore the diffusion time scale τ*_D_* in the early stage of recording is still small. This holds even for the samples with multifunctional monomer species only. The results in Figure 13 therefore suggest that in the reflection recording case most of the drop in ∆*n*_1_ can be attributed to different diffusion attenuation due to the different emerging photopolymer network densities (rigidities) at different monomer mixing ratios.

From the experimental results described above we decided for subsequent simulations to identify the diffusion attenuation factor α as:

(37) $$\alpha =\frac{G_{photocured}}{G_{0}}$$

As we now know the relation of the diffusion attenuation factor α versus the mixing ratio of mono- and multifunctional monomers via the relation shown in Figure 13 we simulated the spatial frequency response of a standard formulation versus a formulation with a majority of monofunctional monomers for red laser (633 nm) saturation recording. We used all the other model parameters we identified by fitting the dosage response curves for doing this.

**Figure 14:**
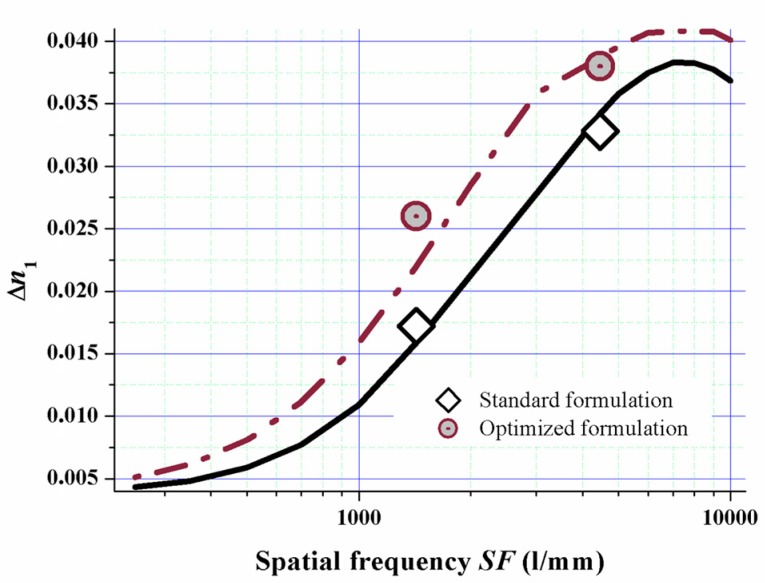
Experimental values of ∆*n*_1_ (symbols) and simulated ∆*n*_1_ response versus spatial frequency *SF* for a standard formulation (solid line) and a formulation with a majority of monofunctional components (dashed line) [51]. The typical error of experimentally determined ∆*n*_1_ values is about ±0.001.

The model almost quantitatively predicts the experimental values as can be clearly seen from Figure 14. This proves the suitability of the model for formulation optimization. By implementing this fully detailed reaction-diffusion model for Bayfol^®^ HX film, we identified and rationalized a formulation design strategy to tune spatial frequency response of the photopolymer to a desired property profile.

## 6. Mechanical Modulus from Soft Rubber to Hard Rubber

As can be imagined from the previous section the modulus *G*_0_ in the unrecorded state should be reasonably large to guarantee sufficient mechanical stability for recording and web-coating [53] and also α should stay close to 1 to allow a fast diffusion of monomers until high conversion has been achieved and by this a high ∆*n*_1_ over wide range of *SF* can be realized (see Figure 14). Also a small *G*_0_ in the unrecorded state gives an inherent tack that allows easy lamination to the recording stack with excellent optical and mechanical coupling. In Bayfol^®^ HX type photopolymers *G*_0_ is usually in the range of 0.1 to 0.5 MPa. This corresponds to the property profile of a soft rubber.

**Figure 15:**
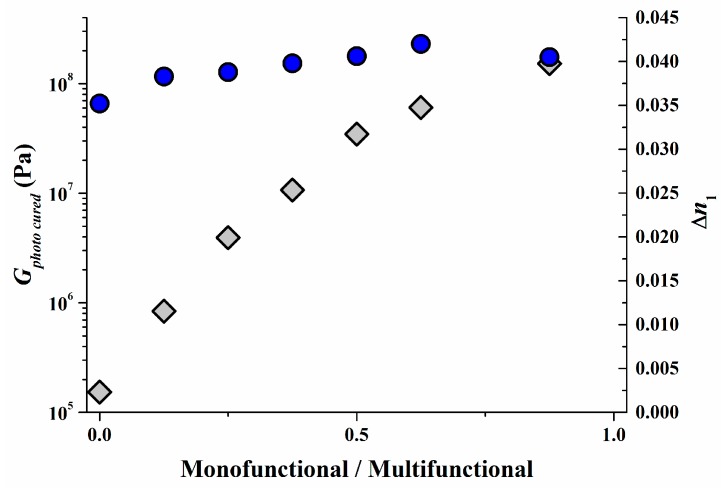
∆*n*_1_ (**right** *y*-axis, blue circles) and shear modulus *G_photo cured_* after flood cure (**left** *y*-axis, grey squares) versus monomer mixing ratio (fraction of the multifunctional monomer increasing from 0 to 1).

However in certain applications the mechanical modulus *G_photo cured_* in the fully recorded or photo-cured should be as high as possible for example to better sustain high pressures and/or high temperatures during vHOE integration into the final device. As we have shown already in Figure 14, by adapting the mixing ratio of mono- and multifunctional monomers *α* can be varied by 2.5 decades. By this a *G_photo cured_* typical for very hard rubbers or typical for soft thermoplastic materials can be achieved. However for the Bayfol^®^ HX type photopolymer depicted in Figure 14 the increase of α goes on the cost of the ∆*n*_1_. By using an adapted matrix [54] this loss of ∆*n*_1_ by increasing α can be completely avoided as it is shown in Figure 15. In this case, we increase α by 3 decades boosting *G_photo cured_* to >100 MPa without any significant variation of ∆*n*_1_.

## 7. Bleaching Process

After recording the vHOE it is mandatory to cure any residual writing monomers and bleach any residual dye from the initiator system. For this usually a flash of UV/VIS light can be used. However care has to be taken not to use a too high dosage of UV light as there is always the threat to induce yellowing in the substrate and/or photopolymer layer. In the laminated state oxygen inhibition of the bleaching of the residual dye is very well suppressed and the necessary dosage for excellent results is lowest. In this section, we describe how to optimize the bleaching without additional yellowing.

For this we use a combination of narrow banded UV LEDs and VIS LEDs to bleach and cure the photopolymer. Compared to a metal halide bulb this combination allows for (i) a rather homogeneous illumination; (ii) a control of the UV wavelength and (iii) a control of UV dosage and VIS dosage separately. Furthermore, the UV LED and VIS LED combination can be switched on and off as required by the step-and-repeat process whereas such a cycling would dramatically decrease the lifetime of a metal halide bulb or mercury bulb.

**Figure 16:**
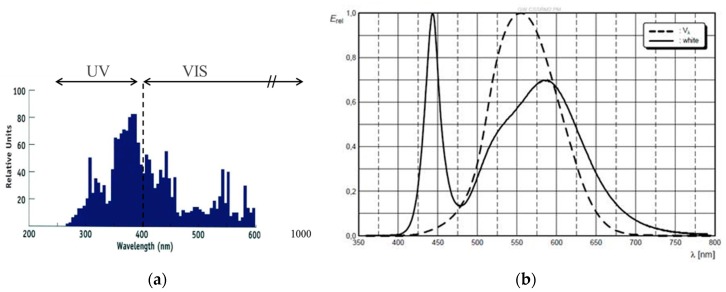
(**a**) UV light-curing flood lamp spectral distribution from a standard metal-halide bulb (DYMAX 2000; source: www.dymax.com). Indicated are also the UV and VIS radiation components as defined by the sensitivity of the detectors (Gigahertz-Optik–X11 Optometer; UV: UV-3719-4; VIS: RW-3705-4); (**b**) White LED Philips ClearFlood BVP650 [55].

The spectrum of a standard metal halide bulb (DYMAX 2000; VIS: 50 mW/cm^2^ and UV: 52 mW/cm^2^) is shown in Figure 16 together with the separation into UV and VIS radiation parts as defined by the sensitivity of the detectors we use for intensity measurements. Obviously this spectrum differs from a UV LED and VIS LED spectrum (see also Figure 16 for the VIS LED spectrum). Most prominently is the broader UV part in the spectrum of the metal halide bulb that extent well below the 365 ± 5 nm line of the UV LED.

The intensity itself and the dosage, i.e., the exposure time times the intensity, govern the bleaching result. A better bleaching result is defined as a higher transmission in the visible spectral region (~400–800 nm) as measured in a spectrometer with the film laminated onto a glass slide. Even though both parameters may be carefully optimized for each Bayfol^®^ HX film grade, as for every organic material we find: the more intensity and dosage is applied during bleaching the better the result. However, if the UV wavelength is too short (<365 nm) and the dosage is too high yellowing of the material starts and yellowing increases with increasing UV dosage.

**Figure 17:**
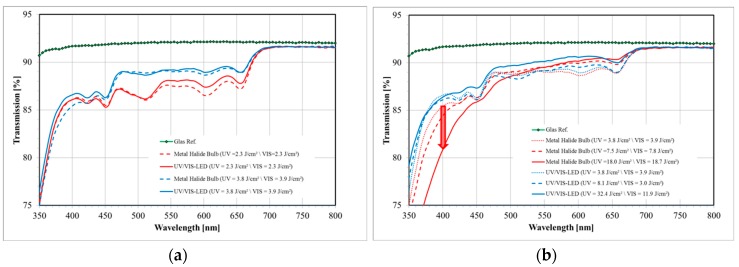
Comparison of bleaching results in which a metal halide bulb and a UV LED and VIS LED combination are used. (**a**) For the same dosage the performance of both curing systems is similar. Moreover, a higher dosage usually gives a better bleaching result; (**b**) Yellowing effect: due to the UV-B part of the metal halide bulb (cf. Figure 16) the transparency in the deep blue (black arrow) is significantly decreased. This effect is not apparent if the UV LED and VIS LED combination is used since this bleaching unit only has a single UV line radiation spectrum at 365 ± 5 nm [56].

This is exemplarily shown in Figure 17a in which the UV LED and VIS LED combination is compared to the metal halide bulb: In both cases the higher dosage yields a better transmission in the visible region. Furthermore, it can be seen that the performance of both devices is similar if the same dosage is applied.

To illustrate the yellowing effect we bleached a certain Bayfol^®^ HX film grade with a TAC substrate using both curing systems. As can be seen in Figure 17b the transparency in the deep blue (~400 nm) is decreased with increasing dosage of the metal halide bulb radiation. In contrast, the transparency around 400 nm stays relatively constant if the UV LED and VIS LED is used for bleaching. This prominent effect appears even though the applied UV dosage by the UV LED and VIS LED combination is roughly a factor of 2 larger compared to the metal halide bulb. Moreover, it can be clearly seen that in the case of the UV LED and VIS LED combination the higher UV dosage helps to increase the overall transmission in the visible spectral region (~400–800 nm).

## 8. How to Mass Produce Volume Holographic Optical Elements with Bayfol^®^ HX Film

For the commercialization of vHOEs simple and robust mass production schemes are of utmost importance. Therefore at the end of this review we would like to touch on this aspect too. In [55,56] we reported about a contact copy process from a so-called master vHOE. In this copy process Bayfol^®^ HX film is laminated to this master vHOE. A single scanning laser line copies the phase hologram of the master vHOE into the copy film. The copy film is moved in a step and repeat process through the replication line. To record a master vHOE in Bayfol^®^ HX film without being bound to size limitations by laser power limits we described in [57] a holographic printer.

First we review the main topics of computer generated volume holographic optical elements generated in a printer and which can be used as master vHOEs. Second we outline the main topics of the replication procedure indicated above for Bayfol^®^ HX film.

### 8.1. Mastering-Overcoming Size and Laser Power Limitations

A major bottleneck for applications using large size vHOEs has been the master hologram recording which, if recorded in traditional full aperture mode, needs expensive, large and high precision optical equipment and high power laser with long coherence length to achieve the minimum needed intensities in the plane of the recording medium. Furthermore, the recording setup, especially the object path needs to be rearranged.

A large master vHOE can be recorded alternatively using only standard size and off the shelf optics and low power lasers, if they are combined with an *x*, *y*-translation stage [58,59]. Small sub-holograms (Hogels) are recorded and stitched next to each other to generate the large vHOE. The Hogels can be recorded by using a physical object generating the object beam or which is much more flexible by a spatial light modulator (amplitude or phase SLM) in the object beam. The usage of a programmable SLM gives us a very high flexibility to generate various types of vHOEs in the same optical and mechanical setup. In the following we review a holographic printing system and one example of a printed vHOE in Bayfol^®^ HX film, both given in [57].

### 8.2. Experimental Setup of the HOE Printer

**Figure 18:**
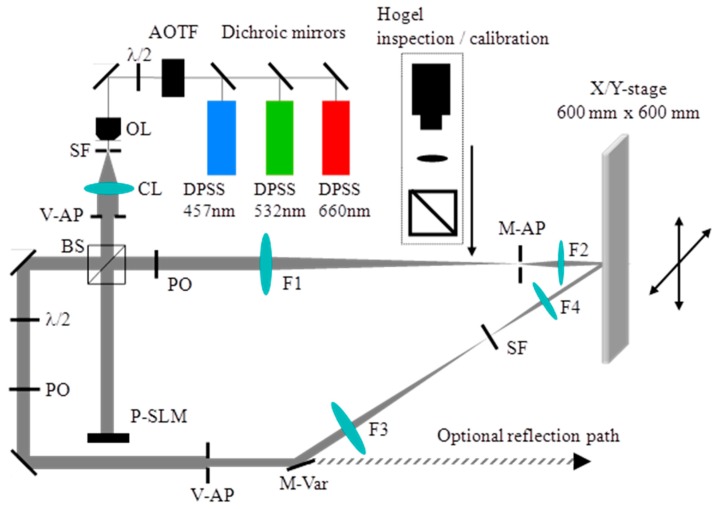
Setup of the holographic printer in which a 600 mm × 600 mm large CGH can be exposed [57].

The holographic printing system described here records functional transmission type vHOEs. The *x*, *y* translation stage that we used allows the recording of vHOE sizes up to 600 mm × 600 mm. Figure 18 shows the optical and mechanical layout of the printing system. A phase only SLM is used to form the object beam of a Hogel. More details on the components used are given in [57].

### 8.3. Application Example: Off-Axis vHOE Lens

One example for a functional vHOE is a holographic off-axis transmission lens.

#### 8.3.1. Recording Scheme

**Figure 19:**
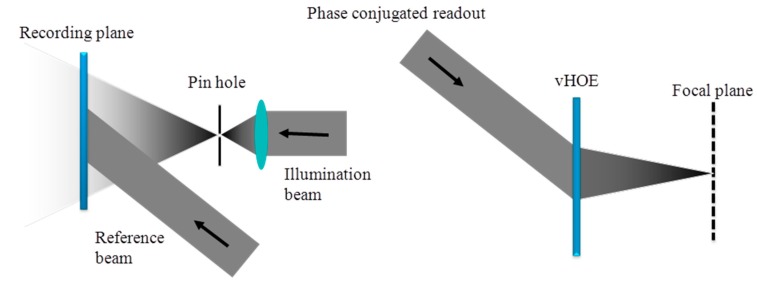
Analog recording scheme for an off axis transmission lens using a pin hole (**left**) as an object; The phase conjugated readout of the off axis transmission lens to reconstruct the real image of the pin hole (**right**) [57].

Figure 19 (left) shows the full aperture recording setup for a holographic off-axis transmission lens. In this case, the object to record is realized by a very small circular aperture (pin hole) at the location of the desired focal point and a plane reference wave is used. Using the phase conjugated reference beam for replay will reconstruct the real image of this vHOE, which is the focal point of this off-axis lens, as shown in Figure 19 (right). For vHOEs of the size of a few centimeters this recording process is simple and easy to setup. However for vHOEs of larger size this recording process will impose immediately severe challenges. To form the large plane reference wave respective lenses or collimating mirrors have to be at least as large as the vHOE. Therefore those components will be heavy and expensive. A homogeneous power distribution (no Gaussian) of the recording beams in the plane of the vHOE is mandatory to achieve equal diffraction efficiencies across the vHOE. Therefore the recording beams have to be expanded much wider than the vHOE area itself, which leads to a huge waste of power and by this a low power density or long exposure times. On the other hand a minimum power density is needed to overcome the oxygen inhibition (see Section 5). Within this tradeoff state of the art single-mode, high power lasers limit the practical vHOE size to a few 10 centimeters. Also very stable and actively fringe locked recording setups are needed for long exposure time.

**Figure 20:**
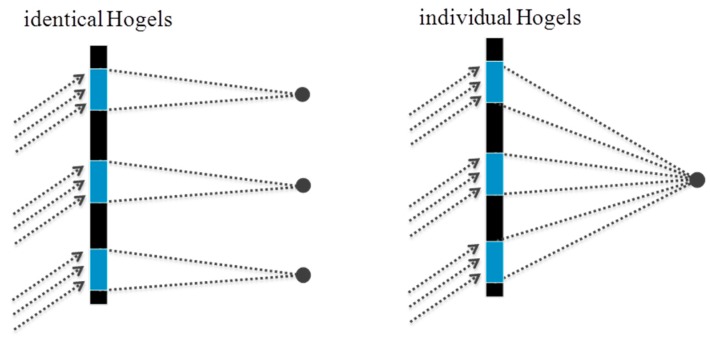
(**left**) A vHOE stitched with identical Hogels. The same function at a different position is reconstructed; (**right**) A vHOE stitched with individual Hogels. Each Hogel is corrected with respect to its position on the vHOE, so that a single focal point is reconstructed [57].

On the other hand it is also possible to “stitch” such a vHOE of an off axis lens sequentially. The phase function needed in the object beam is generated by starting the design algorithm with a small circle. On the left side of Figure 20 it is shown how it looks like if each Hogel would be encoded with the identical phase function of this circle.

To reconstruct a single focal point, for each Hogel a deflecting phase function has to be added to the phase function of the small circle. The deflection has to be chosen with respect to the position of the Hogel on the vHOE and the desired focal length (see Figure 20 (right)).

#### 8.3.2. Experimental Verification

**Figure 21:**
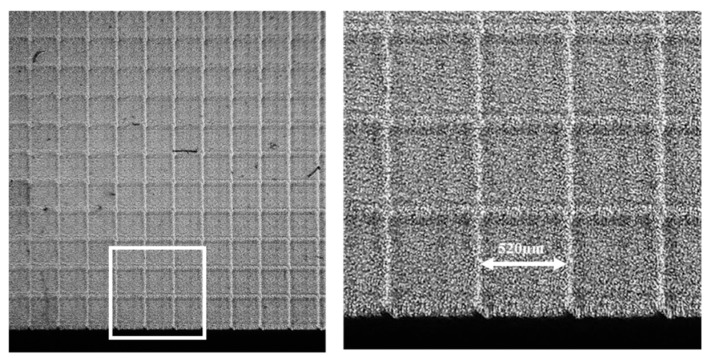
Magnified image of a 60 × 60 Hogel array forming an off-axis holographic lens. The camera system was focused on the vHOE plane and the Hogels are reconstructed “On Bragg” with the reconstruction beam [57].

As shown in [57] a vHOE comprised of a matrix of 60 × 60 Hogels was recorded. Using low power single frequency DPPS lasers with output powers of ~100 mW, total power densities of 100 mW/cm^2^ can be easily achieved at a Hogel size of 520 µm × 520 µm. An exposure time of 200 ms delivers the total dosage of 20 mJ/cm^2^. Besides the exposure time the movement and settling time of the *x*, *y*-translation stage contributes most to the total printing time of ~25 min. The individual deflecting phase function for each Hogel was selected such that a focal distance of 300 mm was reached. A magnified image of the vHOE plane, taken from its reconstruction direction (see Figure 21) reveals that perfect alignment of the Hogels was achieved and that they cover the complete vHOE area.

**Figure 22:**
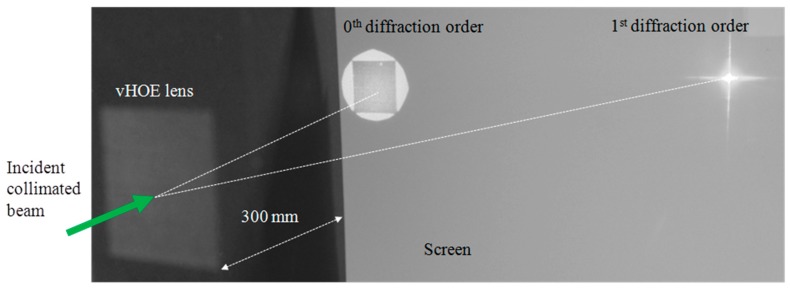
The printed holographic off-axis lens is reconstructed with a plane wave incident under 30° (“On Bragg”) [57]. As the plane wave is larger than the vHOE size a dark square in the 0th diffraction order appears. At the focal distance of 300 mm the 1st diffraction order forms the focal point.

The functionality of the printed off-axis transmission lens was demonstrated by illuminating it at 30° (On Bragg) with a plane wave with a diameter larger than the size of the vHOE (Figure 22 (left)). The incident power on the vHOE can be calculated by multiplication of the average incident power density of ~0.7 mW/cm^2^ with the vHOE size of ~9.7 cm^2^ to be 6.8 mW. The 1st diffraction order of the vHOE at a distance of 300 mm is shown in Figure 22 (right). The size of the focal point was measured to be ~3 mm^2^ and the power in the focal plane was measured to be 5.3 mW. By this, a real diffraction efficiency of 78% could be estimated including Fresnel losses and other losses.

### 8.4. R2R Replication of Volume Holographic Optical Elements

**Figure 23:**
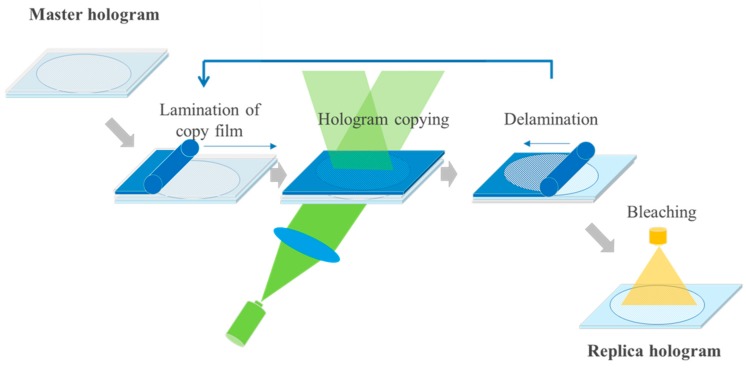
Contact copy scheme of a transmission vHOE. From left to right: The copy film is laminated onto the master vHOE; a laser beam illuminates the master vHOE “On Bragg”, whereby the master vHOE acts as a beam splitter that generates the object beam. This object beam interferes with the residual 0th order beam behind the master vHOE, thereby creating the interference pattern that is recorded into the copy film; downstream within a few seconds the dark reaction is finished; the copy film is delaminated from the master vHOE and protected by re-masking with a cover film; bleaching and fixing is done as last step further downstream [55].

In [55,56] we explained the contact copy process for mass replication of vHOEs in a step and repeat process as stated already above and outlined the mechanical construction and optical beam shaping of the installed replication line. Figure 23 displays the scheme of process steps for a transmission vHOE. The respective scheme for a reflection vHOE is shown in [55,56].

Holographic replication reduces the complexity of the optical setup. The laser beam handling is simplified, since only one beam needs to be managed. The master creates an object beam that propagates to the copy film and interferes with the illumination beam. Properly designed, a master—copy process is beneficial from a vibrational point of view allowing for a more robust process.

#### 8.4.1. Roll-To-Roll (R2R) Mechanics

**Figure 24:**
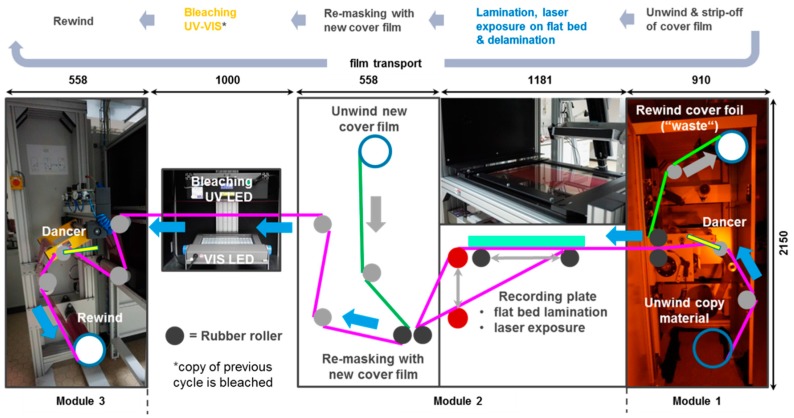
Film handling diagram of the R2R machine with dimensions in millimeters and process description [55].

The R2R film handling is realized by a custom made machine that was designed and built with standard winding components according to the author’s specifications by a machine builder company. Figure 24 shows the film handling diagram of the R2R machine that has a web width of 30 cm. A more detailed description of its individual modules of the replication line is given in [55,56].

The sequence of steps before laser exposure consists of unwinding the copy film, release of the copy film’s cover foil and lamination of the copy film’s photopolymer surface on a glass plate optically bonded with the master hologram. Optical quality lamination is possible on the flat-bed master plate of 500 mm × 300 mm size due to the inherent tackiness of the free photopolymer film surface. Moreover, this tackiness also leads to a self-cleaning of the master plate from adhering dust particles after a few cycles. As shown in Figure 24, we laminate the copy film to the lower side of the master plate. After lamination the line scan exposure is performed. Then, the copy film is de-laminated from the master in a controlled way. For this the rubber roll used for lamination is moved backwards horizontally and the web tension automatically releases the copy film directly at the rubber roll’s contact line from the master plate. After that the film is moved forward preparing the next step-and-repeat cycle. The photopolymer surface of the copy film—now comprising the copied vHOE—is re-masked with a new cover foil. Subsequently, the protected copy film is moved through the bleaching station and finally is rewound to a new core.

#### 8.4.2. The Laser Scanning Module

**Figure 25:**
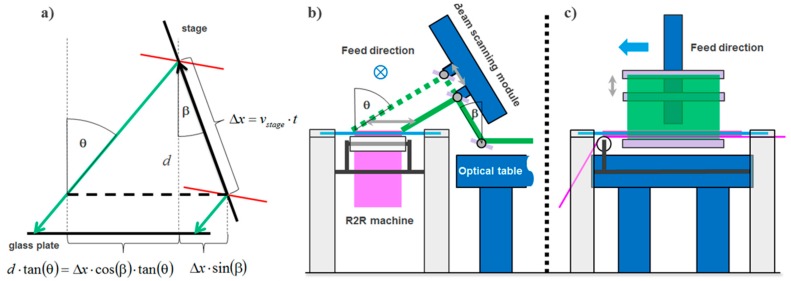
Detailed layout of the laser scanning module. (**a**) Geometrical model; (**b**,**c**) sketch of module. The incidence angle θ can be varied between 20° and 60°, whereas the slanted mount of the stage β is fixed to 20° [55].

The laser scanning module moves a plane wave with a highly elliptical intensity profile at a fixed incidence angle across the recording plate at the time when the copy film is laminated to the copy plate. The layout is shown in Figure 25.

As can be seen from Figure 25b the laser scanning direction is set perpendicular to the copy film feed direction. A major benefit is that there is more space available for the scanning mechanism and optics to shape the wave front of the plane wave to the highly elliptical intensity profile.

The scanning module deflects the plane wave upwards by a rectangular front surface mirror. The holder of this first front surface mirror is mounted on the optical table that supports the beam and wave front shaping optics. A second front surface mirror is mounted on a linear translation stage, whereas this linear translation stage is held by a custom made frame. This frame is mounted on an optical table while not being connected to the frame of the R2R machine. The slanted mount of the linear stage guarantees best balance between achievable scan length at a large range of possible incidence angles and height of the scanning module. To adjust the incidence angle the second mirror is mounted on a rotational plate, which is connected to a motorized rotary stage. Incidence angles θ of the scanning beam towards the surface normal of the master between 20° and 60° can be realized in this setup. Therefore, a high flexibility in usable master vHOE designs can be accommodated. Both front surface mirrors have a size of 130 mm × 430 mm and are made of N-BK7 glass comprising a protected Ag coating.

#### 8.4.3. Optics for Beam Shaping and Wave Front Shaping

**Figure 26:**
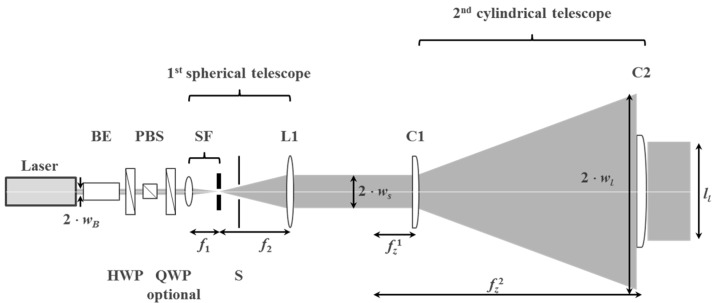
Detailed layout of the beam and wave front shaping optics. The total path length from the spatial filter (SF) to C2 is about 3 m. If the optional QWP is set, the HWP and PBS are removed [55].

The module transforms the circular output laser beam of diameter 2 × *w_B_* = 2.25 mm into a plane wave with a highly elliptical intensity profile with a minor axis of 2 × *w_s_* = 40.9 mm and a major axis of 2 × *w_l_* = 954.5 mm (Figure 26). Along the major axis the power is ~70% of the center power if a 400 mm wide aperture is used. In the module the laser beam is first circularly expanded and cleaned by a spatial filter SF. Second, this circular divergent beam is collimated by a first lens L1 to a 1/*e*^2^ diameter *d*_3_ = 2 × *w_s_* of the short edge of the elliptical intensity profile. Third, a concave cylinder lens C1 expands the beam along the horizontal direction to a desired length at a certain distance. Fourth, a second “large” cylinder lens C2 re-collimates the beam along the horizontal axis to a 1/*e*^2^ radius *w_l_* that corresponds to an edge-to-center power ratio of 70% along a long edge of length *l_l_* = 400 mm. The components for the beam expansion and wave front shaping optics were chosen such that all fits on an optical table of 1 m × 4 m size and that the *NA* of the lenses, especially the cylinder lenses, are kept small (<0.2) to avoid complex and costly lens surface shapes corrected for spherical aberrations. Detailed specifications of the optics are listed in [55,56].

#### 8.4.4. Bleaching Module

**Figure 27:**
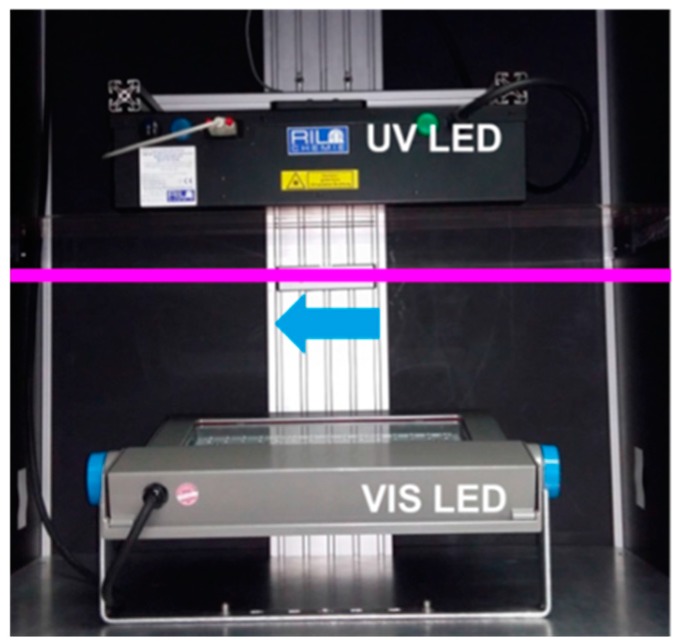
Layout of the bleaching module. The intensity of the various light sources at the copy film plane can be varied by sliding the light sources up and down [55].

The bleaching module has to provide a suitable spectrum and intensity (see Section 7) to irreversibly bleach the residual initiator dyes and cure residual writing monomers. To avoid the damage of the substrate and the recorded photopolymer layer (e.g., deformation by too high heat, yellowing by too high UV dosage, …) an optimum result could be obtained by a combination of UV and VIS LED modules arranged like in Figure 27. The details of the components are listed in [55,56].

#### 8.4.5. Dosage Response of the Contact Copy Process

In order to compare the scanning contact copy process with a static exposure, we measured dosage response curves of vHOE copies recorded in Bayfol HX^®^ films, i.e., the relative diffraction efficiency η of the copy as function of the applied dosage *D* during recording. In a static exposure this is usually done by varying the exposure time at a constant intensity *I*_0_ during recording. In our machine this can be implemented by using the computer controlled shutter. The impinging intensity for any specific position on the copy recording plate can be measured, e.g., by using a power detector head with a circular mask having a 1 mm diameter pinhole (which is beneficial due to the relatively narrow Gaussian envelope along the short edge).

For the scanning copy process a dependency of dosage *D* on the scanning speed *v* needs to be derived from the machine parameters. Details can be found in [55].

**Figure 28:**
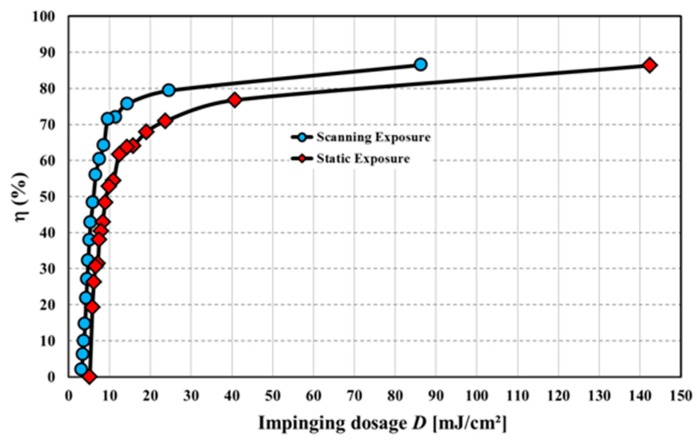
Evidence of independence on vibrations in the contact copy process: using the scanning exposure (**circles**) a similar dosage response curve as in the static exposure (**diamonds**) is achieved for various scanning speeds. Thus, vibrations do not deteriorate the diffraction efficiency η as in the copy with spacing case [55].

As an example we show in Figure 28 the results of the copy of a digital printed master vHOE (plane wave 0°/plane wave 30°) that has a diffraction efficiency of 40%. Thus, the beam ratio *BR* for the copy is 0.67. We applied scanning speeds *v_stage_* between 2 mm/s and 55 mm/s in the scanning case and used 0.52 to 14.19 s for the static exposure. *I_measured_* was 11.5 mW/cm² in both cases.

As we used the contact copy process the dosage response for the scanning process indeed follows that of the static exposure case. This proves the viability of the presented contact copy process and its suitability for mass production of vHOEs.

## 9. Conclusions

We describe the Chemistry and the Physics of a new class of holographic photopolymers (Bayfol^®^ HX film) that allow a pure photonic processing to manufacture volume holographic optical elements (vHOEs). Holographic recording is done by laser interference recording. Several variants of recording schemes are shown including digital master hologram recording and mass photonic replication. The photochemistry to build a light sensitive material is explained in detail and the chemical response is based on the reaction driven diffusion process. Consequences on the recording conditions are explained.

vHOEs in photopolymers are a key enabler to many new applications. While vHOEs do not represent a standalone product and need to work in conjunction with other components, including light source, spatial light modulators and alike, they need to be customized towards specific optical properties. This film media technology is an enabler towards those needs and links itself to classic film processing. Major consumer applications are in development like augmented reality glasses and head-up displays that will change the way we interact with the world in the future. The combination of Physics and Chemistry has been the driver for the development of these materials, which now will bridge towards consumer electronics, the automotive industry and beyond.

## Figures and Tables

**Table 1:** Oxidation potentials *E_ox_*, rates of polymerization *R_p_* and final conversions *C* of the borate salts used in the photo-polymerizing resin with Rose Bengal (**RB**) or Safranine O (**SF**) as dye [35].

Borate	*E_ox_* (V/SCE)	RB *R_p_* (M^−1^∙s^−1^)	*C* (%)	SF *R_p_* (M^−1^∙s^−1^)	*C* (%)
**1**	0.23	1.20	80	0.50	61
**2**	0.38	0.76	77	1.2	67
**3**	0.49	0.16	72	1.8	74
**4**	0.72	0.046	63	1.5	72
**5**	0.74	0.020	49	0.94	70
**6**	0.81	0.0038	15	1.3	71

**Table 2:** Quenching rate constants *k_q_^S,T^* (M^−1^∙s^−^^1^) for the excited states of the dye in acetonitrile and the corresponding Gibbs free energies ∆*G_et_* (eV) [35].

Borate	*k_q_^S^*/∆*G_et_^S^* ^1^RB	*k_q_^T^*/∆*G_et_^T^* ^3^RB	*k_q_^S^*/∆*G_et_^S^* ^1^SF	*k_q_^T^*/∆*G_et_^T^* ^3^SF
**1**	1.8 × 10^9^/−0.94	2.2 × 10^6^/−0.57	1.8 × 10^10^/−1.56	1.8 × 10^10^/−0.98
**2**	1.6 × 10^9^/−0.79	1.5 × 10^6^/−0.42	1.8 × 10^10^/−1.41	1.3 × 10^10^/−0.83
**3**	^a^/−0.68	^a^/−0.31	1.2 × 10^10^/−1.30	8.2 × 10^9^/−0.72
**4**	8.0 × 10^8^/−0.45	6.3 × 10^5^/−0.08	2.0 × 10^10^/−1.07	6.8 × 10^9^/−0.49
**5**	7.3 × 10^8^/−0.43	7.7 × 10^5^/−0.06	1.4 × 10^10^/−1.05	3.8 × 10^9^/−0.47
**6**	4.8 × 10^8^/−0.36	4.3 × 10^5^/−0.01	1.6 × 10^10^/−0.98	9.4 × 10^8^/−0.40

^a^ Not measurable due to insolubility at high concentrations of **3**.

**Table 3:** Total radical quantum yield φ*_R_* formed by reaction of 1.5 × 10^−2^ M of borate salts and Rose Bengal or Safranine O excited states in the photo-polymerizing resin [35]. In brackets: maximum refractive index modulation as obtained in holography recording.

Borate	RB φ*_R_*	SF φ*_R_*
**1**	0.376	0.285
**2**	0.323	0.284
**3**	-	0.285
**4**	0.201 (0.0078)	0.285 (0.0078)
**5**	0.227 (0.0090)	0.284 (0.0085)
**6**	0.154 (0.0065)	0.284
